# Sex-dependent impact of *Il6* deficiency in Parkinson’s disease mice

**DOI:** 10.1016/j.gendis.2025.101986

**Published:** 2025-12-15

**Authors:** Fangzheng Chen, Yufei Duan, Mengze Wang, Zhaolin Liu, Jiayin Zhao, Guangchun Fan, Yongtao He, Hongtian Dong, Xiaoshuang Zhang, Rong Fang, Yunhe Zhang, Xin Yan, Chenye Shen, Xiwen Tang, Yuanyuan Ma, Mei Yu, Renyuan Zhou, Jian Fei, Fang Huang

**Affiliations:** a Department of Translational Neuroscience, Department of Urology, Jing’an District Centre Hospital of Shanghai, National Key Laboratory of Brain Function and Diseases and MOE Frontiers Center for Brain Science, Institutes of Brain Science, Fudan University, Shanghai 200032, China; b School of Life Science and Technology, Tongji University, Shanghai 200092, China; c Shanghai Engineering Research Center for Model Organisms, Shanghai Model Organisms Center, INC., Pudong District, Shanghai 201203, China

**Keywords:** IL-6, MPTP, Neuroinflammation, Parkinson’s disease, rIL-6

## Abstract

Parkinson’s disease (PD) is a prevalent neurodegenerative disorder accompanied by neuroinflammation. Many studies have demonstrated that interleukin-6 (IL-6) exhibits both anti-inflammatory and pro-inflammatory effects in the central nervous system, yet its role in PD remains controversial. In this study, *Il6*^*−/−*^ (knockout), *Il6*^+/−^, and wild-type mice were utilized to investigate the impact of IL-6 on the pathology of MPTP- or α-Synuclein^A53T^-induced PD mice. Our findings revealed that *Il6* deficiency exacerbated motor dysfunction in both female and male mice. MPTP intoxication resulted in earlier and more extensive injuries to the dopaminergic system and heightened glial reaction in the nigrostriatal pathway of female *Il6*^*−/−*^ mice compared with male *Il6*^*−/−*^ mice, which only displayed more severe dopaminergic neuronal loss at 7 days after MPTP administration. Toxic α-Synuclein overexpression in the substantia nigra region caused earlier motor dysfunction and aggravated dopaminergic neurodegeneration in female *Il6*^*−/−*^ mice. In *Il6*^+/−^ mice, MPTP-induced depletion of dopaminergic nerve fibers was unaffected, although astrocyte activation was attenuated. Moreover, intraperitoneal administration of recombinant IL-6 (rIL-6) partially ameliorated MPTP-induced motor dysfunction and striatal dopaminergic terminal depletion in both wild-type and knockout mice. Our findings underscore the crucial role of IL-6 in the inflammatory pathology of PD, highlighting sex-dependent differences, and suggest that rIL-6 holds potential promise for PD therapy.

## Introduction

Parkinson’s disease (PD) is the second-most common neurodegenerative disorder after Alzheimer’s disease.^1^ The prevalence of PD is approximately twice as high in men compared with women, although women tend to experience more severe symptoms in the advanced stages of the disease.^2^, ^3^, ^4^ PD is characterized by the degenerative loss of dopaminergic neurons in the nigrostriatal pathway.^5^^,^^6^ Previous studies have shown that neuroinflammation, induced by glial reaction, exacerbates nigrostriatal degeneration.^7^, ^8^, ^9^, ^10^ Although significant sex-specific differences in PD are acknowledged, the underlying mechanisms remain unclear.

Recent studies have shown a strong association between interleukin-6 (IL-6) and PD.^11^^,^^12^ Elevated IL-6 levels have been detected in the serum and cerebrospinal fluid of PD patients,^11^^,^^13^^,^^14^ in 1-methyl-4-phenyl-1,2,3,6-tetrahydropyridine (MPTP)-induced PD mouse models,^15^ and in 1-Methyl-4-pheny1-pyridinium (MPP^+^) -induced SH-SY5Y and BV2 cell lines.^16^ However, IL-6 plays a dual role, exhibiting both pro-inflammatory and neuroprotective effects in the central nervous system.^17^^,^^18^ For example, IL-6 can induce neuronal death by enhancing NMDA receptor-mediated excitotoxicity,^19^ promote toxic neuronal iron accumulation,^20^ and contribute to α-Synuclein-induced neurodegeneration.^21^ Up-regulation of IL-6 exacerbates dopaminergic neuronal loss in 6-hydroxydopamine (6-OHDA)-induced PD rats,^22^ and blocking IL-6 signaling prevents astrocyte-induced neurodegeneration in an iPSC-based PD model.^23^ Conversely, IL-6 has been shown to exert neuroprotective effects in the central nervous system.^24^, ^25^, ^26^, ^27^ For example, IL-6 secreted by astrocytes treated with low concentrations of lipopolysaccharide protected dopaminergic neurons,^28^ and increased IL-6 levels led to reduced pro-inflammatory factor release in MPTP-induced PD mice following rapamycin treatment.^29^ Additionally, recombinant IL-6 (rIL-6) was found to attenuate MPP^+^-induced toxicity in primary dopaminergic neurons of mice and rats.^30^^,^^31^ Thus, the role of IL-6 in PD is complex and sometimes even contradictory.

IL-6 signals through three pathways: the classical pathway (IL-6 binds to the membrane receptor mIl6R and the membrane protein gp130), the trans-signaling pathway (IL-6 binds to the soluble receptor sIl6R and the membrane protein gp130), and the trans-presentation pathway (like that Il6 binds to the dendritic cell-presented IL-6R and gp130 on the T-cell).^32^ Downstream signaling of IL-6 involves the Janus kinase–signal transducer and activator of transcription (JAK–STAT), RAS–mitogen-activated protein kinase (RAS–MAPK), phosphatidylinositol 3-kinase–protein kinase B (PI3K–AKT), and nuclear factor kappa B (NF-κB) pathways.^33^ Notably, there is crosstalk between the pathways of IL-6 and estrogen.^34^ Estrogen can inhibit IL-6 production.^35^ Individuals with the GG/GG genotype combination of the *IL6* G-174C and the *ERβ* G-1730A single-nucleotide polymorphisms (SNPs) have an increased risk of developing PD.^20^ Research has quantified plasma estradiol levels in female wild-type (WT) mice (1017 ± 175 ng/mL) and female *Il6*^*−/−*^ mice (697 ± 205 ng/mL),^36^ suggesting that *Il6* deletion can reduce estradiol levels in female mice. Notably, estrogen is known to protect dopaminergic neurons in PD.^37^^,^^38^ Bolin et al found that *Il6* deficiency increased the vulnerability of the dopaminergic system in a single-dose MPTP-induced male PD mice,^39^^,^^40^ whereas the role of *Il6* deficiency in female mice and the related mechanisms remain to be elucidated.

In this study, we analyzed the behaviors and nigrostriatal degeneration in *Il6*^*−/−*^ (knockout/KO) and WT mice of both sexes following an acute regimen of MPTP administration. To further explore the primary relationship between IL-6 and the chronic PD progression, α-Synuclein^A53T^ overexpression model was established in both *Il6*^*−/−*^ and WT female mice. The results indicate that *Il6* deficiency exacerbates PD pathology, with a more pronounced and earlier damaging effect observed in female PD mice. Additionally, we investigated the interventional effects of intraperitoneal administration of rIL-6 on PD pathology, which demonstrated certain protective effects in PD mice.

## Materials and methods

### Animals

Adult *Il6*^*−/−*^, *Il6*^*+/−*^, and WT mice, all on a C57BL/6 background, were sourced from the Shanghai Model Organisms Center (Shanghai, China). The mice were housed in groups under a 12-h/12-h light/dark cycle, with unrestricted access to food and water. All experiments involving these mice were performed in compliance with the guidelines of the Institutional Animal Care and Use Committee of Fudan University, Shanghai Medical College. Care was taken throughout the study to minimize potential harm or stress to the animals.

### MPTP and rIL-6 administration in mice

To establish an acute PD mouse model, mice were intraperitoneally injected with MPTP (Sigma, USA) at a dose of 15 mg/kg, administered four times at 2-h intervals. Normal saline was used as a control.

For rIL-6 treatment, mice received intraperitoneal injections of 500 ng rIL-6 (MedChemExpress, USA) 30 min before the first MPTP injection and 1 h after the fourth injection. Additional doses of 500 ng rIL-6 were given on the first and second days following MPTP administration, for a total of four rIL-6 injections. For AAV α-Synuclein^A53T^-induced PD mice, three intraperitoneal injections of 500 ng rIL-6 were administered from day 33 to day 35 after virus injection.

### Stereotaxic injection for α-Synuclein^A53T^-induced PD model

AAV2/9 was used as a viral vector to deliver the cassette hα-Syn overexpressing the A53T mutation of human α-Synuclein [(rAAV-SYN -SNCA(A53T)-WPRE-bGH pA, AAV α-Synuclein^A53T^), 1.29 × 10^13^ v.g/mL, BrainVTA Co., Ltd., China]. AAV2/9-CMV-EGFP (AAV GFP, 5.12 × 10^12^ v.g/mL, BrainVTA Co., Ltd., China) was used as the control. Three-month-old female WT and *Il6*^*−/−*^ mice were stereotaxically injected in the bilateral substantia nigra (SN) regions with AAV α-Synuclein^A53T^ or AAV GFP, respectively.

The apparatus of stereotaxic injection (RWD, China) was used for the injection under anesthesia of isoflurane. Targeting the SN region of coordinates anteroposterior (AP) 3.15 mm, mediolateral (ML) 1.25 mm, and dorsoventral (DV) 4.50 mm, 200 nL of virus was injected at a rate of 80–100 nL/min and held still for 10 min. The animals were then placed in the heated blanket to recover. Behavior tests were performed weekly starting from 14 days post-injection until 35 days, and then the mice were euthanized for sample collection 4 days later.

### Rearing test

Mice were individually placed in a transparent beaker (8 cm in diameter, 18 cm in height) and observed for 3 min. During this time, the number of times they reared, standing on their hindlimbs with their forelimbs resting against the beaker wall, was recorded.

### Pole test

Mice were positioned head upwards at the top of a wooden pole (80 cm in height). The turning time, descent time, and total time were documented. Before MPTP administration, each mouse underwent a two-day training period. The formal test was conducted three times to obtain an average time.^41^

### Wire hanging test

A suspended wire stretched between two 60 cm high wooden poles served as the apparatus, with small wooden planks on either side acting as platforms. Mice were placed on the wire, suspended by their forelimbs, at its midpoint. A point was awarded each time a mouse successfully reached a platform, while a point was deducted for each fall to the ground. The total score was calculated over 3 min, starting with a base score of 10 points.

### Open field test

Each mouse was individually placed at the center of an open field apparatus measuring 40 × 40 × 40 cm and observed for a duration of 5 min or 10 min. Behavior parameters, including total distance traveled, average speed, frequency, and the number of entries into the central area, were recorded and analyzed using Noldus EthoVision XT software (Noldus, Netherlands).^42^

### Rotarod test

The rotarod test was used to assess motor learning and coordination.^43^ Mice were trained for 5 min each day over 2 consecutive days at a constant speed of 10 rpm. For the testing phase, mice were placed on the rotarod apparatus as the speed gradually accelerated from 4 rpm to 40 rpm within 5 min. The total distance was recorded for 3 trials. To maintain cleanliness, both the rod and chambers were cleaned using 75% ethanol between trials.

### Tape removal test

Tape removal test, also named adhesive removal test, was conducted following the previous procedure.^44^ Briefly, each mouse was individually placed at the center of an open field box measuring 15 × 20 × 30 cm for a habituation period of 1 min. The animal was gently removed from the testing box, and one adhesive tape strip was applied with equal pressure on each animal’s paws. Once the adhesive tape strip had been placed, the mouse was replaced in the testing box and timed. Both the latency to feel the tape and the time taken by the animal to remove the tape were recorded for 3 min. Between trials, the testing box was cleaned using 75% ethanol.

### Protein extraction and Western blotting

Striatum tissues were lysed using 200 μL of RIPA lysis buffer (Beyotime Biotechnology, China) supplemented with 1 × protease and 1 × phosphatase inhibitors (Epizyme, China). Protein concentrations were measured with a BCA assay kit (Beyotime Biotechnology, China). A total of 30 μg of protein from each sample was resolved on a 12.5% SDS-PAGE gel and subsequently transferred onto a polyvinylidene fluoride (PVDF) membrane.

The membrane was blocked with 5% skim milk at room temperature for 1 h, followed by incubation with primary antibodies at room temperature for 1 h and then at 4 °C overnight. The primary antibodies used included mouse anti-tyrosine hydroxylase (TH) antibody (1:1000; Abcam, USA), rabbit anti-glial fibrillary acidic protein (GFAP) antibody (1:1000; Proteintech, China), mouse anti-β-actin antibody (1:1000; Epizyme, China), mouse anti-α-Synuclein antibody (syn211) (1:500; Abcam, USA), rabbit anti-α-Synuclein antibody (1:2500; HUABIO, China), and rabbit anti-IL-6 antibody (1:1000; HUABIO, China). After thorough washing with Tris-buffered saline-Tween 20 detergent (TBST), the membrane was incubated with a fluorescent secondary antibody (1:20,000; LI-COR, USA) at room temperature for 1 h.

Western blot images were acquired using the Odyssey Infrared Imaging System (LI-COR, USA), and protein band intensities were quantified using ImageJ software (v1.8.0; National Institutes of Health, USA).

### High-performance liquid chromatography

Striatum tissues were homogenized in cold 0.4 M perchloric acid (HClO_4_) at a volume of 10 μL per milligram of tissue. The homogenate was centrifuged at 10,000 rpm at 4 °C for 10 min, and the supernatant was collected for analysis. The levels of dopamine (DA), serotonin (5-HT), 3,4-dihydroxyphenylacetic acid (DOPAC), homovanillic acid (HVA), and 5-hydroxyindoleacetic acid (5-HIAA) were measured using high-performance liquid chromatography with an ESA system (ESA, USA).^45^

### Immunofluorescence and immunohistochemistry staining

Mouse brains were fixed in 4% paraformaldehyde for 24 h, followed by dehydration in 20% and 30% sucrose solutions for 24 h each. The brains were then embedded in an optimal temperature cutting (OCT) compound (Sakura Tissue-Tek, Japan) and sectioned into 30 μm coronal slices using a cryostat (Leica, Wetzlar, Germany).

Immunofluorescence staining: Sections were blocked with phosphate-buffered saline containing 10% goat serum and 0.05% Triton X-100 at 37 °C for 1 h. They were then incubated with primary antibodies at 37 °C for 2 h and then at 4 °C overnight. After washing with phosphate-buffered saline, the slices were incubated with Alexa Fluor-conjugated secondary antibodies (Alexa Fluor 488 and 594, 1:1000; Invitrogen, USA) at 37 °C for 1 h. Images were acquired using a Nikon AIR-MP confocal microscope (Japan).

For immunohistochemistry, slices were treated with 0.3% hydrogen peroxide (H_2_O_2_) at room temperature to quench endogenous peroxidase activity. After blocking with phosphate-buffered saline containing 10% goat serum and 0.05% Triton X-100 at 37 °C for 1 h, sections were incubated with primary antibodies at 37 °C for 1 h and then at 4 °C overnight. The following primary antibodies were used: mouse anti-TH (1:1000; Abcam, USA), rabbit anti-GFAP (1:1000; Proteintech, China), and rabbit anti-Iba1 (1:1000; Abcam, USA). After washing, sections were treated with a biotinylated secondary antibody (1:200; Vector Laboratories, USA) and an avidin-biotin peroxidase complex (1:200; Vector Laboratories, USA) at 37 °C for 45 min. Detection was performed using a DAB kit (Vector Laboratories, USA). Images were captured using an Olympus microscope (Japan).

### Cell counting

A Stereo Investigator system (Micro Brightfield, USA) was used to quantify TH^+^ neurons in the substantia nigra pars compacta (SNpc). Six coronal sections, spaced at 10-slice intervals, were analyzed for each mouse, covering the Bregma region from 2.80 mm to 3.80 mm. Neuronal counts were performed in a double-blinded manner.^46^

To enumerate glial cells, four 30-μm-thickness sections per mouse were analyzed. Using ImageJ (v1.8.0), two 400 μm × 400 μm regions were delineated within the striatum, or the SN was manually circumscribed. The outlined areas were automatically calculated by the software, and cell numbers within these regions were quantified.^47^

### Statistical analysis

Data were presented as mean ± standard error of the mean and were analyzed using Prism 8 (GraphPad Software Inc., USA). Group differences were evaluated using one-way or two-way ANOVA, followed by LSD multiple comparison tests for normally distributed data or Dunn’s test for non-normally distributed data. For data of repeated measures, group differences were evaluated using two-way RANOVA, followed by LSD multiple comparison tests for normally distributed data or Dunn’s test for non-normally distributed data. Statistical significance was defined as *P* < 0.05.

## Results

### *Il6* deficiency exacerbates motor dysfunction in MPTP-induced PD mice of both sexes

**Figure 1:**
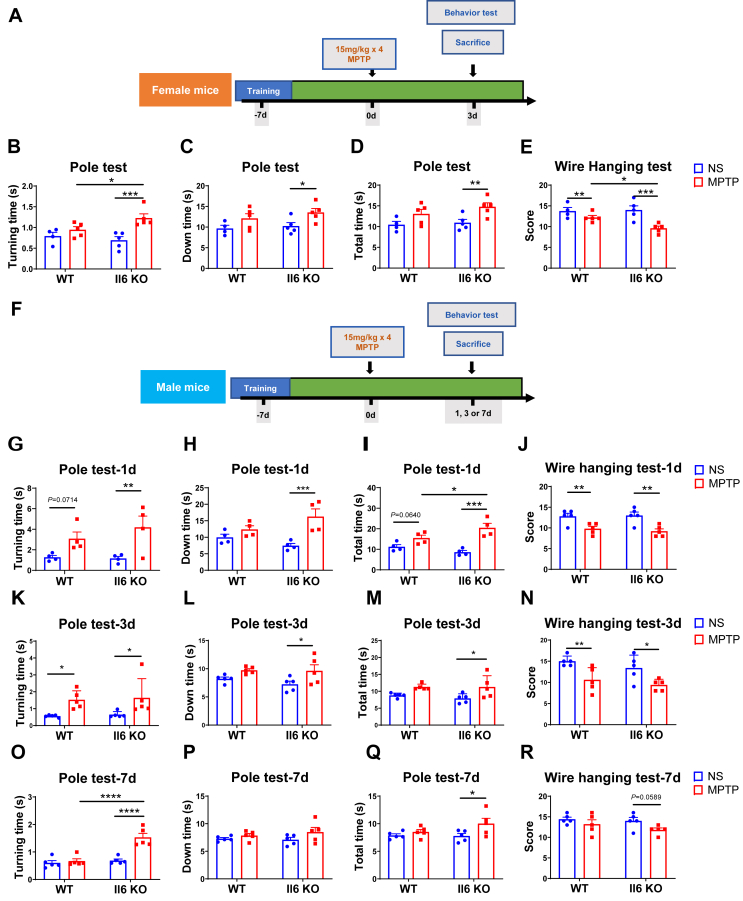
The experimental schedules and the results of behavioral tests. **(A, F)** The flowchart of acute Parkinson’s disease modeling experiments and Parkinson’s disease-related behavioral tests conducted in *Il6* knockout (KO) mice and wild-type (WT) mice of both sexes. **(B**–**D)** The results of the pole test in female mice. Turning time (B), down time (C), and total time (D) were shown. *n* = 4 or 5. **(E)** The score of the wire hanging test in female mice. *n* = 4 or 5. **(G–I, K–M, O–R)** The results of the pole test in male mice at 1 day (G–I), 3 days (K–M), and 7 days (O–R) after MPTP administration. Turning time (G, K, O), down time (H, L, P), and total time (I, M, Q) were shown. *n* = 4 or 5. **(J, N, R)** The score of the wire hanging test in male mice at 1 day (J), 3 days (N), and 7 days (R) after MPTP administration. *n* = 5. Data were analyzed by Two-way ANOVA followed by Fisher’s LSD test for comparisons between groups. ∗*P* < 0.05, ∗∗*P* < 0.01, ∗∗∗*P* < 0.001, and ∗∗∗∗*P* < 0.0001.

Initially, the expression of IL-6 proteins in MPTP-induced PD mice was evaluated by Western blotting. The result revealed that striatal IL-6 protein levels were significantly increased in female MPTP-induced PD mice (Fig. S1A and S1B), whereas male PD mice just displayed a tendency of higher levels of striatal IL-6 proteins (Fig. S1A and S1C). To investigate the sex-specific effect of IL-6 in PD, we then examined PD pathology in both female and male *Il6*^*−/−*^ and WT mice challenged with MPTP. Under physiological conditions, both female and male KO mice showed no significant differences in total moving distance, average speed, or total moving time in the open field test compared with their WT counterparts. However, female KO mice spent less time in the central area and exhibited a remarkable reduction in the ratio of center moving distance to total distance (Fig. S2A). Male KO mice also spent less time in the center, with reduced moving time in the center compared with WT mice. The ratio of center moving time to total moving time was also decreased (Fig. S2B). An acute PD model was established via intraperitoneal injection of MPTP. Behavioral tests related to PD were conducted in female mice at 3 days post-injection (Fig. 1A) and in male mice at 1, 3, and 7 days post-injection (Fig. 1F).

In the pole test, MPTP-treated female WT mice showed no significant differences compared with their normal saline-treated controls. In contrast, MPTP-treated female KO mice exhibited significantly increased turning and climbing down time and total time when compared with their normal saline-treated controls. Moreover, the turning time for MPTP-treated female KO mice was markedly longer than that of WT counterparts (Fig. 1B–D). In the wire hanging test, both genotypes of female mice exhibited obvious impairments, with KO mice scoring lower than WT mice following MPTP administration (Fig. 1E). However, at 1 day post-MPTP injection, both genotypes of male mice spent a longer time to turn around and complete the test. KO mice, but not WT mice, also took a longer time to climb down, and MPTP-treated KO mice spent a longer time to complete the test when compared with their WT counterparts in the pole test (Fig. 1G–I). In the wire hanging test, scores for both genotypes of male mice were significantly reduced compared with their respective controls (Fig. 1J). At 3 days post-MPTP injection, both genotypes of male mice spent a longer time to turn around in the pole test (Fig. 1K–M) and scored low in the wire hanging test (Fig. 1N). However, KO male mice, but not WT mice, spent more time climbing down and completing the pole test (Fig. 1L and M). At 7 days post-MPTP injection, male WT mice displayed normal behavior in both the pole test and the wire hanging test, while male KO mice continued to show impaired performance in these two behavioral tests (Fig. 1O–R). These results suggest that *Il6* deficiency exacerbates MPTP-induced motor dysfunction.

### *Il6* deficiency aggravates dopaminergic damage in the nigrostriatal pathway of mice, with female mice affected earlier than male mice

**Figure 2:**
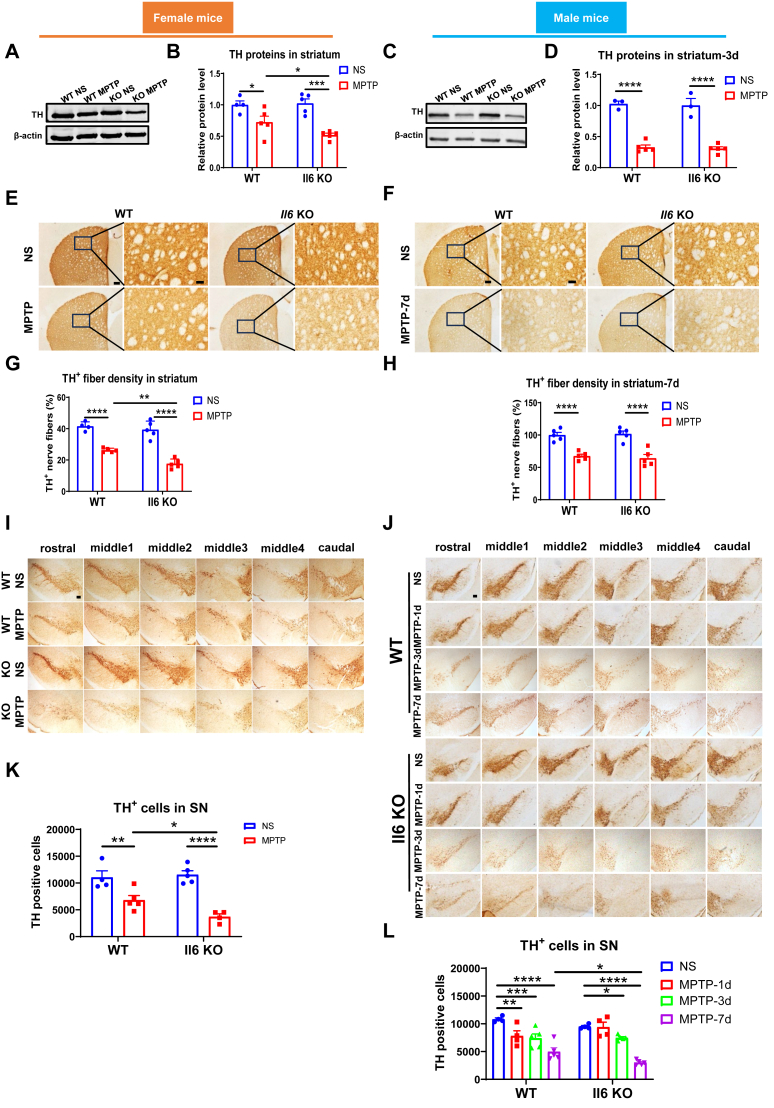
Alterations of the nigrostriatal dopaminergic system after Parkinson’s disease modeling in *Il6* knockout (KO) and wild-type (WT) mice of both sexes. **(A**–**D)** TH protein levels in the striatum of female mice (A, B) and male mice (C, D) at 3 days post-MPTP administration. β-actin served as the loading control. *n* = 3–5. **(E**–**H)** Density of TH-positive nerve fibers in the striatum of female mice (E, G) at 3 days post-MPTP administration (*n* = 4 or 5) and male mice (F, H) at 7 days post-MPTP administration (*n* = 5). Scale bar: 200 μm and 50 μm (zoom). **(I**–**L)** Counting of TH-positive cells in the SNpc of female mice (I, K) at 3 days post-MPTP administration (*n* = 4 or 5) and male mice (J, L) at 1 day, 3 days, and 7 days post-MPTP administration (*n* = 4 or 5). Scale bar: 200 μm. ∗*P* < 0.05, ∗∗*P* < 0.01, and ∗∗∗∗*P* < 0.0001.

MPTP can induce nigrostriatal dopaminergic injuries in mice. Following MPTP intoxication, both female (Fig. 2A and B) and male (Fig. 2C and D) mice of two genotypes exhibited a significant reduction in TH protein levels in the striatum at 3 days post-injection. Notably, only female KO mice showed a more pronounced decrease in TH protein expression compared with WT mice (Fig. 2A and B), but not in male KO mice (Fig. 2C and D). Consistent with these observations, the density of TH-positive nerve fibers in the striatum (Fig. 2E–G) and the number of remaining TH-positive neurons in the SNpc (Fig. 2I–K) were significantly lower in MPTP-treated female KO mice than in their WT counterparts. In male KO mice, a higher loss of TH-positive neurons in the SNpc was observed at 7 days post-injection, but not at 1 day or 3 days post-injection, compared with WT mice (Fig. 2J–L). Male mice of both genotypes exhibited a similar depletion of striatal TH-positive nerve fibers at 7 days after MPTP injection (Fig. 2F–H), and similar reductions of striatal TH proteins at 1 day and 7 days post-MPTP administration (Fig. S3A–S3D).

High-performance liquid chromatography analysis revealed no significant differences in the striatal concentrations of DA, DAPOC, HVA, 5-HT, and 5-HIAA, nor in the ratios of DOPAC/DA, HVA/DA, and 5-HIAA/5-HT between male mice of the two genotypes, with or without MPTP treatment. However, MPTP administration resulted in a dramatic decrease in DA, DAPOC, and HVA concentrations at 3 days post-injection. Furthermore, in MPTP-treated KO mice, the HVA/DA ratio increased significantly, while striatal 5-HT levels decreased compared with normal saline-injected KO mice (Fig. S4). The results indicate that *Il6* deficiency exacerbates MPTP-induced damage to the nigrostriatal dopaminergic system, with female mice affected earlier than male mice.

### *Il6* deficiency exacerbates astrocyte reaction in the nigrostriatal pathway of female mice, but not male mice, following MPTP administration

**Figure 3:**
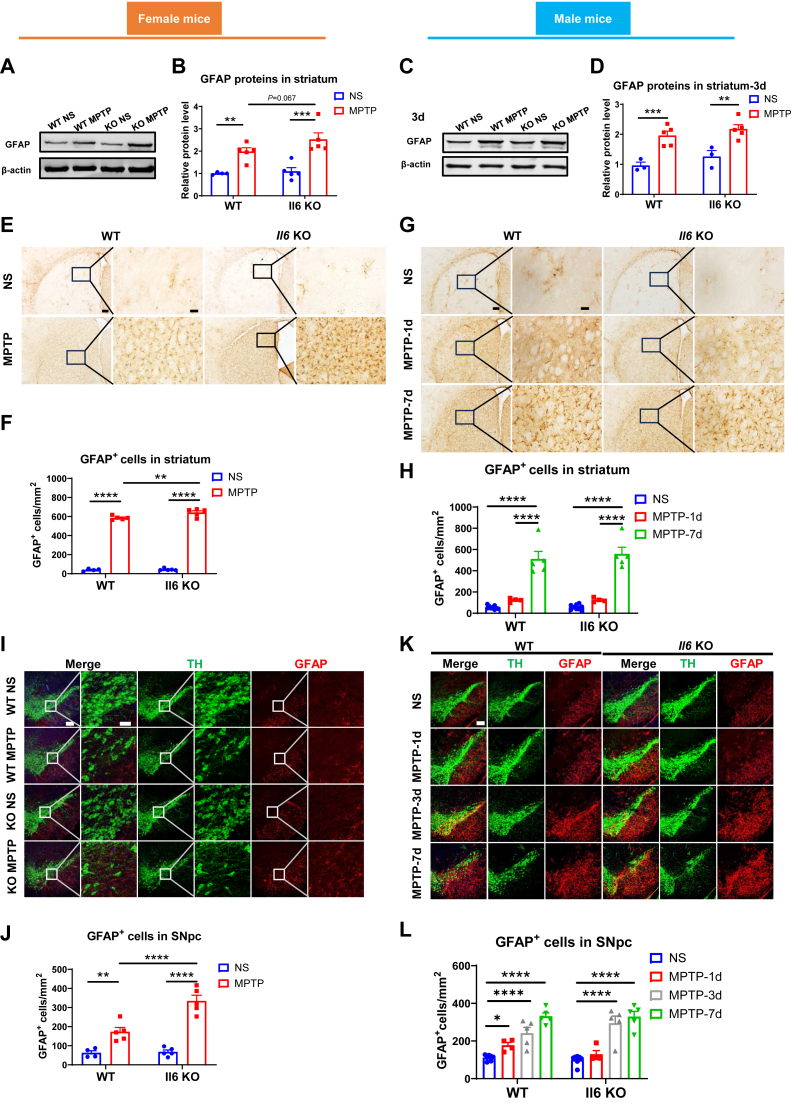
Analysis of astrocyte activation in the nigrostriatal pathway following Parkinson’s disease modeling in *Il6* knockout (KO) and wild-type (WT) mice of both sexes. **(A**–**D)** GFAP protein levels in the striatum of female mice (A, B) (*n* = 4 or 5) and male mice (C, D) (*n* = 3–5) at 3 days post-MPTP administration. β-actin served as the loading control. **(E**–**H)** Immunohistochemical staining and cell counting of GFAP-positive cells in the striatum of female mice (E, F) at 3 days post-MPTP administration (*n* = 4 or 5) and male mice (G, H) at 1 day and 7 days post-MPTP administration (*n* = 5–7). Scale bar: 200 μm and 50 μm (zoom). **(I**–**L)** Immunofluorescence staining and cell counting of GFAP-positive cells in the SNpc of female mice (I, J) at 3 days post-MPTP administration (*n* = 4 or 5) and male mice (K, L) at 1 day, 3 days, and 7 days post-MPTP administration (*n* = 4–7). Representative TH (green) and GFAP (red) images are shown. Scale bar: 200 μm and 50 μm (zoom). Data were analyzed by two-way ANOVA followed by Fisher’s LSD test for comparisons between groups. ∗*P* < 0.05, ∗∗*P* < 0.01, ∗∗∗*P* < 0.001, and ∗∗∗∗*P* < 0.0001.

The brains of PD patients are characterized by a heightened inflammatory state, marked by the reactive activation of astrocytes.^48^ In MPTP-intoxicated female and male mice of both genotypes, GFAP protein levels in the striatum were significantly up-regulated at 3 days post-injection (Fig. 3A–D). In female KO mice, GFAP expression showed a trend towards higher levels compared with WT controls (*P* = 0.067) (Fig. 3A and B), while no differences in GFAP protein levels were observed between MPTP-treated male mice of both genotypes (Fig. 3C and D).

By immunohistochemistry and immunofluorescence staining and cell counting, we found that GFAP-positive cells were significantly increased in the nigrostriatal pathway of MPTP-challenged female mice at 3 days post-injection. This increase was further pronounced in KO mice compared with WT counterparts (Fig. 3E, F, I, J). In contrast, in the striatum of male mice, GFAP-positive cells significantly increased at 7 days, but not at 1 day post-MPTP administration, with no differences between the two genotypes (Fig. 3G and H). Additionally, striatal GFAP protein levels did not change at 1 day post-injection but increased dramatically at 7 days post-MPTP administration (Fig. S3E–S3H). Moreover, in the SNpc of male mice, GFAP-positive cells were notably higher compared with normal saline-treated mice at both 3 and 7 days post-MPTP injection, with no genotype differences observed (Fig. 3K and L). The results suggest that *Il6* deficiency further promotes MPTP-induced reactive activation of astrocytes in the nigrostriatal pathway of female mice.

### *Il6* deficiency exacerbates microglial reaction in the striatum of female mice and in the substantia nigra of male mice following MPTP administration

**Figure 4:**
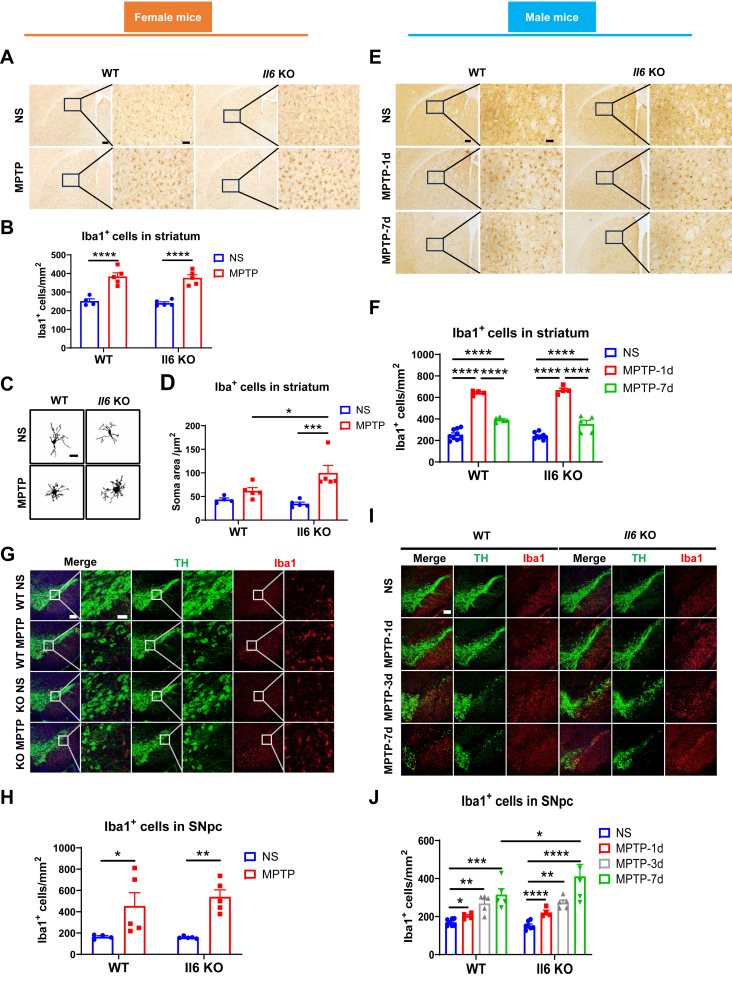
Analysis of microglia activation in the nigrostriatal pathway following Parkinson’s disease modeling in *Il6* knockout (KO) and wild-type (WT) mice of both sexes. **(A**–**D)** Immunohistochemical staining and cell counting (A, B) and morphological analysis (C, D) of Iba1-positive cells in the striatum of female mice at 3 days post-MPTP administration. *n* = 3–5. Scale bar: 200 μm and 50 μm (zoom). **(E, F)** Immunohistochemical staining and cell counting of Iba1-positive cells in the striatum of male mice at 1 day and 7 days post-MPTP administration. *n* = 5–7. Scale bar: 200 μm and 50 μm (zoom). **(G**–**J)** Immunofluorescence staining and cell counting of Iba1-positive cells in the SNpc of female mice (G, H) at 3 days post-MPTP administration (*n* = 4 or 5) and male mice (I, J) at 1 day, 3 days, and 7 days post-MPTP administration (*n* = 4–7). Representative TH (green) and Iba1 (red) images are shown. Scale bar: 200 μm and 50 μm (zoom). Data were analyzed by Two-way ANOVA followed by Fisher’s LSD test for comparisons between groups. ∗*P* < 0.05, ∗∗*P* < 0.01, ∗∗∗*P* < 0.001, and ∗∗∗∗*P* < 0.0001.

Microglia are the primary immune cells within the central nervous system and play a crucial role in the interaction with neurons and astrocytes to perform various functions.^49^ In PD, microglia become activated and migrate to the site of injury to engulf debris and release inflammatory factors. The ionized calcium-binding adapter molecule 1 (Iba1) is recognized as a specific marker for microglia. In the striatum, Iba1-positive cells increased in both female and male mice at 1 day, 3 days, and 7 days post-MPTP injection (Fig. 4A, B, E, F). Further morphological analyses revealed that the microglial soma of MPTP-intoxicated female KO mice was significantly larger than that of female WT mice (Fig. 4C and D). In the SN, immunofluorescence staining showed a dramatic increase in Iba1-positive cells in MPTP-challenged female and male mice of both genotypes compared with normal saline-treated controls (Fig. 4G–J). Notably, in the SNpc of male KO mice, Iba1-positive cells were markedly higher than in male WT mice at 7 days post-MPTP administration (Fig. 4I and J).

### *Il6* heterozygous mice and WT mice show no difference in PD pathology following MPTP administration

To determine whether reduced IL-6 expression affects PD pathology, *Il6* heterozygous mice and WT mice were administered an acute regimen of MPTP. One day later, striatal TH protein levels and glial activation in the nigrostriatal pathway were assessed. Two genotypes of mice showed no marked difference in the decrease of striatal TH proteins (Fig. S5A and S5B), and the increase of Iba1-positive cells both in the striatum (Fig. S5I and S5J) and the SNpc (Fig. S5K and S5L) after MPTP administration. Although the striatal GFAP protein levels (Fig. S5C and S5D) and GFAP-positive cells (Fig. S5E and S5F) did not differ between MPTP-intoxicated and normal saline-treated mice, *Il6* heterozygous mice exhibited significantly lower striatal GFAP protein levels than WT mice post-MPTP challenge (Fig. S5C and S5D). Additionally, GFAP-positive cells increased dramatically in the SNpc of WT but not in *Il6* heterozygous mice (Fig. S5G and S5H). Moreover, MPTP administration activated microglia in the nigrostriatal pathway, with increased numbers observed in both genotypes (Fig. S5I–S5L). No differences in microglial activation were noted between *Il6* heterozygous and WT mice in either the striatum (Fig. S5I and S5J) or the SNpc (Fig. S5K and S5L).

### Striatal proteomic analysis of *Il6* KO and WT mice under physiological conditions and after PD modeling

To determine the protein expression profiles affected by *Il6* deficiency, proteins extracted from the striatal tissues of WT and KO mice of both sexes were utilized for the proteomic analysis. Under physiological conditions, we identified 32 proteins that differed in the striatum of female KO mice compared with their WT counterparts (Fig. S6A). Subsequent Gene Ontology (GO) and Kyoto Encyclopedia of Genes and Genomes (KEGG) enrichment analyses were performed on these differentially expressed proteins (DEPs).^50^ GO analysis revealed that these DEPs were primarily involved in biological processes such as the regulation of immune effector processes, acute inflammatory response, and negative regulation of oxidoreductase activity (Fig. S6B). KEGG enrichment analyses indicated that these DEPs were associated with PD, Huntington’s disease, Alzheimer’s disease, and the estrogen signaling pathway (Fig. S6C).

**Figure 5:**
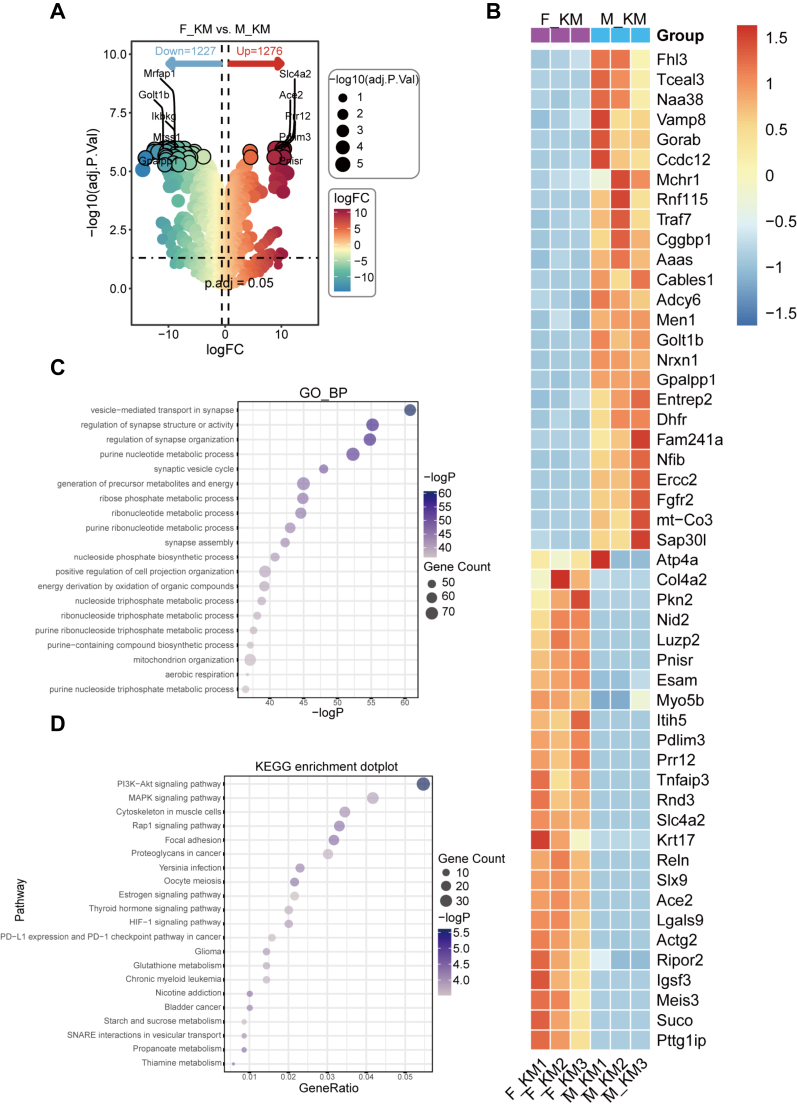
Analysis of differentially expressed proteins in the striatum of female and male *Il6* knockout (KO) mice at 3 days after MPTP administration. **(A)** Volcano plot. **(B)** Heatmap analysis of the top 50 proteins. **(C)** GO analysis. **(D)** KEGG analysis. F_KM: female KO MPTP; M_KM: male KO MPTP.

To investigate whether *Il6* deficiency would change the expression of relative proteins in the striatum following PD modeling, we identified 48 DEPs, including 20 up-regulated and 28 down-regulated proteins in the striatum of female KO mice (Fig. S7A and S7B). GO analysis showed that these DEPs were mainly involved in acute inflammatory responses, dopamine metabolic process, dopamine biosynthetic process, and synaptic transmission (Fig. S7C). KEGG enrichment analysis further demonstrated the significant involvement of DEPs in signaling pathways, including PD, estrogen signaling pathway, MAPK signaling pathway, and PI3K–Akt signaling pathway (Fig. S7D). Besides, 33 DEPs were identified, including 12 up-regulated and 21 down-regulated proteins in the striatum of male KO PD mice (Fig. S8A and S8B). GO analysis showed that these DEPs were mainly involved in dopaminergic synaptic transmission and cell matrix adhesion (Fig. S8C). KEGG enrichment analysis further demonstrated the involvement of DEPs in signaling pathways, including dopaminergic synapse, focal adhesion, neuroactive ligand signaling, hormone signaling pathway, and PI3K–Akt signaling pathway (Fig. S8D). Furthermore, we conducted comparative proteomic analysis of female and male KO mice following MPTP administration and identified 2503 DEPs, including 1276 up-regulated and 1227 down-regulated proteins in the striatum of female KO mice compared with male KO mice (Fig. 5A and B). GO analysis showed that these DEPs were mainly involved in mitochondrion organization, synapse assembly, and regulation of synapse organization (Fig. 5C). KEGG enrichment analysis further demonstrated the involvement of DEPs in signaling pathways, including estrogen signaling pathway, MAPK signaling pathway, and PI3K–Akt signaling pathway (Fig. 5D).

### *Il6* deficiency exacerbates motor dysfunction and aggravates dopaminergic damage of the nigrostriatal pathway in AAV α-Synuclein^A53T^-induced female PD mouse model

Further, we utilized a chronic mouse model of α-Synuclein stress in which AAV carrying disease-associated human mutant α-Synuclein^A53T^ was injected into the mouse SN region to decipher the effect of *Il6* deletion on PD progression (Fig. S9A). During the process of α-Synuclein overexpression, *Il6* KO mice demonstrated greater susceptibility to mutant α-Synuclein toxicity compared with WT counterparts, as evidenced by their early death (Fig. S9B). To determine the temporal progression and magnitude of motor dysfunction induced by AAV α-Synuclein^A53T^, we performed longitudinal behavioral tests from 14 to 35 days post-injection (Fig. S9A). During the testing period, AAV α-Synuclein^A53T^-injected mice with *Il6* deficiency displayed significantly increased down time and total time in the pole test compared wth AAV GFP-injected KO mice, while turning time in AAV α-Synuclein^A53T^-injected *Il6*^*−/−*^ mice showed a significant increase at day 35 compared with WT mice. Moreover, AAV α-Synuclein^A53T^-injected *Il6* KO mice showed significantly longer total time at day 35 compared with AAV α-Synuclein^A53T^-injected WT counterparts, which showed obvious motor deficits starting from day 21 (Fig. S9C–S9E). Additionally, *Il6* KO mice infected with AAV α-Synuclein^A53T^ also displayed fewer rearing events in the rearing test at day 21 and day 28 and a lower score in the wire hanging test from day 14 to day 35 compared with control KO mice (Fig. S9F–S9H). In the rotarod test, only AAV α-Synuclein^A53T^-injected *Il6* KO mice exhibited a significant decrease in total distance at day 28 and day 35 compared with control virus-injected KO mice (Fig. S9I), while WT mice did not show any impairment. In the tape removal test, starting from day 28 to day 35, *Il6* KO mice with AAV α-Synuclein^A53T^ injection took a longer time to remove the tape compared with control KO mice (Fig. S9K), while the time to sense the tape did not show any difference (Fig. S9J). These results further support the notion that *Il6* deficiency exacerbates motor dysfunction in AAV α-Synuclein^A53T^-induced female PD mice.

The human mutant α-Synuclein could be successfully detected in the striatum and SN region of both WT and *Il6* KO mice following viral injection (Fig. S10A–S10C). Whereas, endogenous mouse α-Synuclein proteins showed no significant changes (Fig. S10D and S10E). To investigate whether the effect of *Il6* deficiency in the chronic α-Synuclein PD model would replicate those in the acute MPTP model in terms of the nigrostriatal dopaminergic damage, we analyzed TH proteins in the striatum and TH-positive neurons in the SNpc. The results demonstrated that female mice of both genotypes exhibited a significant reduction in TH protein levels following AAV α-Synuclein^A53T^ injection. Notably, female KO mice showed a more pronounced decrease in TH protein expression compared with their WT counterparts (Fig. S10F and S10G). Consistent with these observations, the number of remaining TH-positive neurons in the SNpc was significantly lower in AAV α-Synuclein^A53T^-injected female KO mice (Fig. S10H and S10I). To know whether *Il6* deficiency exacerbated neuroinflammation in AAV α-Synuclein^A53T^-induced PD model, GFAP and Iba1 protein levels in the striatum of α-Synuclein-stressed female mice were detected by Western blotting. Our results revealed that *Il6* deficiency did not significantly increase the expression of GFAP and Iba1 proteins compared with the WT controls (Fig. S10J–S10M). The results indicate that *Il6* deficiency exacerbates α-Synuclein-induced damage to the nigrostriatal dopaminergic system, but not glial activation in female mice at day 39 after PD modeling.

### Intraperitoneal administration of rIL-6 partially attenuates motor dysfunction in PD mice

**Figure 6:**
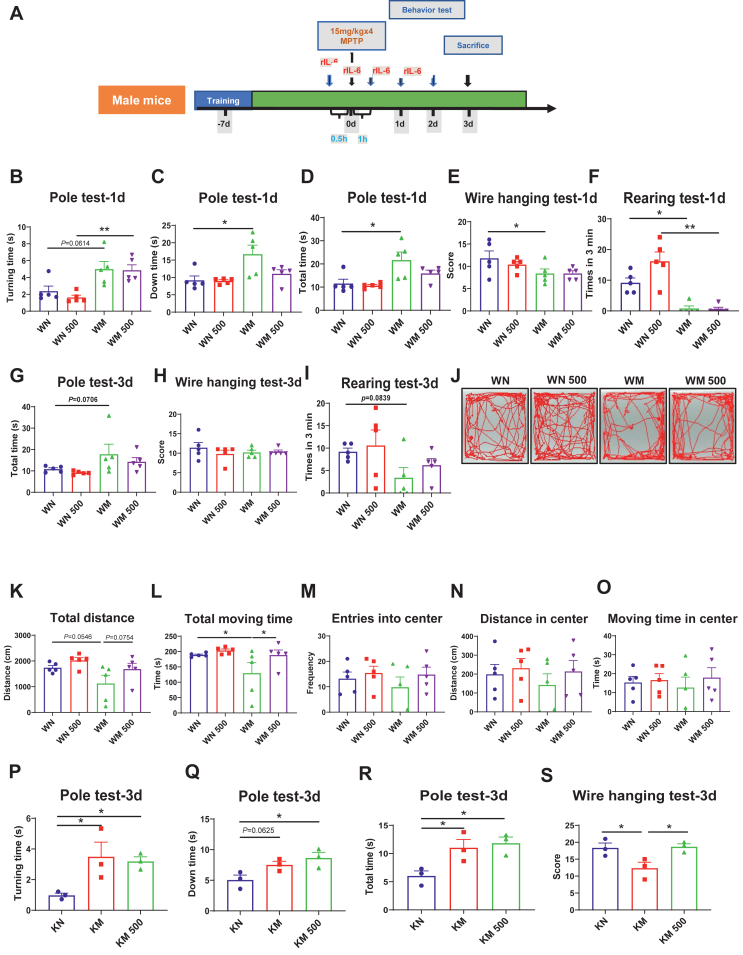
The results of behavioral tests in MPTP-challenged wild-type (WT) and *Il6* knockout (KO) mice with rIL-6 intervention. **(A)** Flowchart of Parkinson’s disease modeling and rIL-6 treatment. **(B–F)** Results of the pole test (B–D), wire hanging test (E), and rearing test (F) in WT mice one day after MPTP administration. **(G**–**I)** Results of the pole test (G), wire hanging test (H), and rearing test (I) in WT mice at 3 days after MPTP administration. **(J**–**O)** Results of the open field test in WT mice at 3 days after MPTP administration. Representative moving trajectories (J), total moving distance (K), total moving time (L), entries into the center (M), distance moving in the center (N), and moving time in the center (O) are shown. *n* = 5. **(P–S)** Results of the pole test (P–R) and wire hanging test (S) in *Il6* KO mice at 3 days after MPTP administration. *n* = 3. ∗*P* < 0.05 and ∗∗*P* < 0.01.

Given that *Il6* KO mice exhibited more severe PD pathology, we explored the potential of rIL-6 as an intervention for PD. rIL-6 has a half-time of 42 min *in vivo*,^51^ and both WT and KO mice were injected intraperitoneally with a dose of 500 ng rIL-6.^52^, ^53^, ^54^ The rIL-6 was administered four times: 30 min before the first injection of MPTP, 1 h after the fourth injection of MPTP, and once daily on the two subsequent days. Behavioral tests related to PD were conducted over the three days following MPTP injection (Fig. 6A). One day after MPTP injection, in the pole test, there was an increase in turning time (*P* = 0.0614), down time, and total time in the WM group (WT mice treated with MPTP alone) compared with the WN group (WT mice treated with normal saline). However, in the WM 500 group (WT mice treated with MPTP and rIL-6), down time and total time did not differ from the WN 500 group (WT mice treated with saline and rIL-6) except for turning time (Fig. 6B–D). In the wire hanging test, the WM group, but not the WM 500 group, displayed a significant reduction in scores (Fig. 6E), while both the WM and WM 500 groups showed markedly lower rearing times in the rearing test (Fig. 6F). Three days after MPTP administration, the WM group exhibited impairments in the pole test (Fig. 6G) and rearing test (Fig. 6I), but not the WM 500 group. Both the WM and WM 500 groups showed no differences from their controls in the wire hanging test (Fig. 6H). In the open field test, the WM group spent less time moving and traveled shorter distances compared with the WN and WM 500 groups (Fig. 6J–L). All experimental groups showed no differences in entries into or moving time and distance in the center area (Fig. 6M−O). In *Il6* KO mice, rIL-6 administration did not alter performance in the pole test (Fig. 6P–R). However, the KM group (KO mice treated with MPTP) had significantly decreased scores compared with the KN (KO mice treated with normal saline) and KM 500 (KO mice treated with MPTP and rIL-6) groups (Fig. 6S). To investigate the dose effect of rIL-6 on PD pathology, a lower dose of 50 ng rIL-6 was also adopted in PD mice (Fig. S11A). Our results demonstrated that the lower dose of rIL-6 did not significantly affect most of the behavioral performance (Fig. S11), except that it did shorten the down time and total time in the pole test 1 day post-injection (Fig. S11C and S11D), indicating an early protective effect of rIL-6 in PD pathology.

### rIL-6 protects dopaminergic nerve fibers, but has a limited effect on glial reaction in the nigrostriatal pathway of PD mice

**Figure 7:**
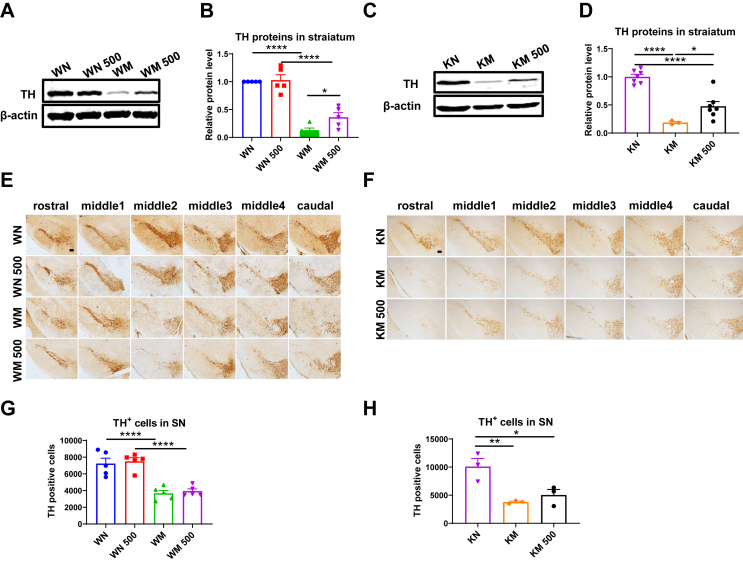
Striatal TH protein expression in MPTP-challenged wild-type (WT) and *Il6* knockout (KO) mice with rIL-6 intervention. **(A**–**D)** Representative Western blot images and quantification of striatal TH proteins in WT mice (A, B) (*n* = 5) and *Il6* KO mice (C, D) (*n* = 3–7) at 3 days post-MPTP administration. β-actin served as the loading control. **(E**–**H)** Counting of TH-positive cells in the SNpc of WT mice (E, G) (*n* = 5) and *Il6* KO mice (F, H) (*n* = 3) at 3 days post-MPTP administration. ∗*P* < 0.05, ∗∗*P* < 0.01, and ∗∗∗∗*P* < 0.0001.

To further assess the impact of rIL-6 on MPTP-induced nigrostriatal dopaminergic damage, we administered four intraperitoneal injections of 50 ng or 500 ng rIL-6. The striatal TH protein expression in the WM 50 group showed an increasing trend compared with the WM group (Fig. S12A and S12B; *P* = 0.0510). 500 ng rIL-6 did not alter striatal TH protein expression or the number of TH-positive neurons in the SNpc of WT mice compared with the saline-treated group (Fig. 7A, B, E, G). Following MPTP administration, both striatal TH protein levels and the number of TH-positive neurons in the SNpc decreased significantly in WT and *Il6* KO mice. However, rIL-6 treatment significantly up-regulated TH protein levels in MPTP-intoxicated WT mice compared with the WM group (Fig. 7A and B), and similarly in KO mice versus the KM group (Fig. 7C and D). Additionally, KO mice in the KM 500 group exhibited more TH-positive cells in the SNpc compared with MPTP-treated KO controls (Fig. 7F–H), whereas no significant difference in TH-positive neuronal loss was observed between the WM and WM 500 groups (Fig. 7E–G). We also conducted Western blotting analysis to measure the expression levels of apoptosis-related proteins Bax and Bcl2, and Iba1 and GFAP proteins in the striatum of WM and WM 50 groups. The results demonstrated that the 50 ng of rIL-6 did not change the expression levels of these proteins (Fig. S12C–S12J). These findings suggest that rIL-6 may partially alleviate MPTP-induced damage to dopaminergic nerve fibers in both WT and *Il6* KO mice.

**Figure 8:**
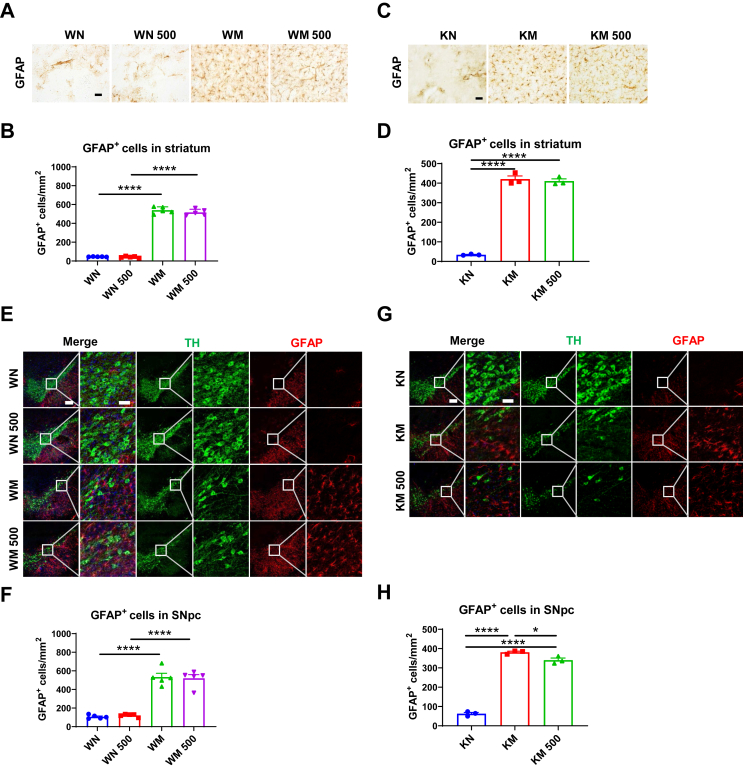
Astrocyte reactivation in the nigrostriatal pathway of MPTP-challenged wild-type (WT) and *Il6* knockout (KO) mice with rIL-6 intervention. **(A**–**D)** Immunohistochemical staining and cell counting of GFAP-positive cells in the striatum of WT mice (A, B) (*n* = 5) and *Il6* KO mice (C, D) (*n* = 3) at 3 days after MPTP administration. Scale bar: 50 μm (zoom). **(E**–**H)** Immunofluorescence staining and cell counting of GFAP-positive cells in the SNpc of WT mice (E, F) (*n* = 5) and *Il6* KO mice (G, H) (*n* = 3) at 3 days after MPTP administration. Representative TH (green) and GFAP (red) images are shown. Scale bar: 200 μm and 50 μm (zoom). ∗*P* < 0.05 and ∗∗∗∗*P* < 0.0001.

We further investigated whether intraperitoneal injection of rIL-6 could affect MPTP-induced activation of glial cells in the nigrostriatal pathway. Our results demonstrated that in the striatum of both WT and *Il6* KO mice, rIL-6 did not alter the number of GFAP^+^ cells in saline-treated or MPTP-treated mice compared with their respective controls (Fig. 8A–D). In the SN, astrocyte numbers were significantly increased in both WM and KM groups compared with their respective saline controls (Fig. 8E–H). However, there was no significant difference in astrocyte numbers between the WM and WM 500 groups (Fig. 8E and F), whereas astrocytes in KM 500 mice were significantly reduced compared with KM mice (Fig. 8G and H). Therefore, rIL-6 appears to mitigate MPTP-induced astrocyte activation only in the SN of KO mice.

Following MPTP administration, microglial activation in the striatum of WT and *Il6* KO mice was evidenced by a significant increase in cell numbers. However, the administration of rIL-6 did not affect Iba1-positive cells in the nigrostriatal pathway (Fig. S13). Overall, intraperitoneal administration of rIL-6 did not influence MPTP-induced microglial activation in the nigrostriatal pathway of WT and *Il6-*deficient mice.

Moreover, to test whether intraperitoneal injection of rIL-6 could affect AAV α-Synuclein^A53T^-induced motor dysfunction, we conducted behavior tests after three doses of 500 ng rIL-6 injection starting at 33 days post-virus injection (Fig. S14A). rIL-6 intervened *Il6* KO mice showed better behavioral performance, as indicated by a decreased tendency of down time (Fig. S14C), shorter turning time (Fig. S14B), and total climbing time (Fig. S14D) in the pole test, and the increased total distance in the rotarod test (Fig. S14H) compared with AAV α-Synuclein^A53T^-injected KO counterparts. However, no significant differences were detected in the rearing test, wire hanging test, and tape removal test (Fig. S14E–S14G, S14I, S14J). To investigate whether rIL-6 could protect against dopaminergic neuronal loss and neuroinflammation in the nigrostriatal pathway, we examined the striatal TH, GFAP and Iba1 protein levels, and found that compared with mice in KA group (AAV α-Synuclein^A53T^-injected KO mice treated with normal saline), the expression of TH proteins of mice in KA 500 group (AAV α-Synuclein^A53T^-injected KO mice treated with 500 ng rIL-6) was significantly increased (Fig. S14K, S14L), GFAP and Iba1 protein levels did not differ between KA and KA 500 groups (Fig. S14K, S14M–S14O). Therefore, rIL-6 could alleviate AAV α-Synuclein^A53T^-induced PD pathology in KO mice.

## Discussion

In this study, we investigated the impact of *Il6* deficiency on PD-like pathology in both female and male mice. Under physiological conditions, both sexes exhibited anxiety-like behavior. *Il6* deficiency exacerbated motor dysfunction in both female and male PD mice. Female KO mice experienced a more severe depletion of dopaminergic nerve fibers following MPTP administration. Correspondingly, the loss of dopaminergic neurons in the SNpc was more pronounced in female KO mice at 3 days post-injection, while male mice showed a more significant reduction in dopaminergic neurons at 7 days post-injection. These findings suggest that *Il6* deficiency exacerbates PD pathology in a sex-specific manner.

Neuroinflammation plays an important role in the progression of PD.^49^^,^^55^^,^^56^ We analyzed the activation of glial cells in the nigrostriatal pathway and found that female KO mice exhibited a significant increase in nigrostriatal astrocytes compared with female WT mice under MPTP induction, while *Il6* deletion did not affect MPTP-induced microglial activation. In male mice, *Il6* deletion did not impact MPTP-induced activation of astrocytes and microglia in the striatum. However, in the SN, *Il6* deletion led to more severe microglial activation at 7 days after MPTP administration. Thus, *Il6* deletion exacerbated glial activation in PD mice in a sex-specific manner. Notably, MPTP-intoxicated *Il6* heterozygous mice did not recapitulate this enhanced glial response.

Our study showed that *Il6* deficiency led to earlier and exacerbated motor dysfunction, and aggravated damage to the nigrostriatal dopaminergic system in α-Synuclein ^A53T^ overexpression-induced female PD mice. In such a chronic PD model, female KO mice exhibited no significant difference in the striatal GFAP or Iba1 proteins compared with female WT mice. This might be due to the relatively short duration (39 days) of α-Synuclein^A53T^ expression. However, the lack of human non-mutant α-Synuclein overexpression control is one of the limitations of this study.

The incidence of PD is closely linked to sex, with a higher prevalence in males than in females. Reports suggest that IL-6 interacts with estrogen, potentially offering protection against PD. *Il6* deletion inhibits estradiol production in female mice.^3^^,^^57^ Pre-treatment with 17β-estradiol and membrane-impermeable estrogen (E2-BSA) was neuroprotective against MPP^+^-induced dopaminergic neuronal toxicity. This neuroprotective effect was significantly reduced by the PI3 kinase inhibitor, Wortmannin.^57^ Proteomic analysis of female KO mice in the MPTP group revealed 48 DEPs compared with WT counterparts. GO and KEGG analyses identified the involvement of the MAPK pathway and the PI3K–Akt pathway. Besides, KEGG enrichment analysis further demonstrated the involvement of DEPs in these two signaling pathways in female KO mice compared with male KO mice under MPTP administration. The MAPK family proteins (*i.e.*, p38, c-JNK, and ERK) are involved in the stress-activated response.^58^ Previous studies have demonstrated that ERK phosphorylation activates Nurr1 and its related factors, thereby regulating TH expression.^59^ The PI3K–Akt pathway plays a crucial role in various cellular processes. It has been revealed that AKT and phosphorylated AKT are significantly decreased in the SNpc of PD patients.^60^ Targeting PI3K–Akt or MAPK on a mouse model of PD results in the suppression of neuroinflammation and neuronal apoptosis in the striatum and SN, leading to neuroprotection against PD.^61^^,^^62^ Therefore, the MAPK pathway and the PI3K–Akt pathway might be involved in the aggregated PD pathology in female mice with *Il6* deficiency. Moreover, compared with male KO mice under MPTP administration, KEGG enrichment analysis in female KO mice further demonstrated the involvement of DEPs in the estrogen signaling pathway, indicating a potential sex-dependent role of IL-6 in PD pathology. We acknowledge that directly manipulating estrogen signaling in our models with receptor agonists or antagonists would strengthen sex-dependent mechanisms, and such a limitation is a focus of deep future investigation.

Given these findings, we investigated the role of rIL-6 in PD mice. Administration of rIL-6 improved behavioral performance in MPTP-treated WT and *Il6* KO mice or α-Synuclein^A53T^-stressed female *Il6* KO mice to some extent. Striatal TH protein levels were significantly up-regulated in MPTP plus rIL-6-treated WT and KO mice or α-Synuclein^A53T^ plus rIL-6-treated female KO mice compared with their MPTP-treated counterparts or AAV α-Synuclein^A53T^-injected KO controls. Notably, only in KO mice treated with MPTP plus rIL-6 was astrocyte activation in the SN significantly reduced compared with MPTP-treated KO controls. These results demonstrate that intraperitoneal injection of rIL-6 significantly protects against MPTP or α-Synuclein^A53T^-induced damage to the dopaminergic nerve fibers in both WT and *Il6* KO mice. Given that rIL-6 can partially cross the blood–brain barrier^51^ and has a protective effect on midbrain dopaminergic neurons *in vitro*,^42^ it is hypothesized that a small amount of rIL-6 may enter the brain of PD mice and exert neuroprotective functions. However, the mechanisms remain to be further elucidated. Long-term impact of IL-6 deficiency on α-Synuclein aggregation, and isolating central versus peripheral IL-6 effects, warrant further studies.

In conclusion, *Il6* deficiency differentially affected PD pathology in female and male mice. Intraperitoneal injection of rIL-6 could effectively mitigate PD pathology in WT and KO mice (Fig. S15).

## CRediT authorship contribution statement

**Fangzheng Chen:** Methodology, Data curation, Conceptualization. **Yufei Duan:** Formal analysis, Data curation. **Mengze Wang:** Methodology. **Zhaolin Liu:** Formal analysis. **Jiayin Zhao:** Methodology. **Guangchun Fan:** Data curation. **Yongtao He:** Visualization. **Hongtian Dong:** Visualization. **Xiaoshuang Zhang:** Resources. **Rong Fang:** Methodology. **Yunhe Zhang:** Software. **Xin Yan:** Resources. **Chenye Shen:** Software. **Xiwen Tang:** Methodology. **Yuanyuan Ma:** Software. **Mei Yu:** Software. **Renyuan Zhou:** Supervision. **Jian Fei:** Supervision. **Fang Huang:** Supervision.

## Ethics declaration

All the animal experiments were conducted in accordance with the guidelines of the Institutional Animal Care and Use Committee of Fudan University, Shanghai Medical College.

## Data availability

All data are available upon contact with the corresponding author.

## Funding

This work was supported by the 10.13039/501100001809National Natural Science Foundation of China (No. 32271003), the 10.13039/501100003399Science and Technology Commission of Shanghai Municipality, China (No. 24141901200), the Shanghai Municipal Science and Technology Major Project (China) (No. 2018SHZDZX01), and ZJLab, the Shanghai Center for Brain Science and Brain-Inspired Technology, the Innovative Research Team of High-Level Local University in Shanghai, China.

## Conflict of interests

The authors declared no competing interests.
