# Supplementary material for: Sex-dependent impact of Il6 deficiency in Parkinson’s disease mice
Source: Genes Dis. 2025 Dec 15;13(4):101986. doi: 10.1016/j.gendis.2025.101986 (PMC13099946; doi:10.1016/j.gendis.2025.101986)
Supplement: Multimedia component 1 [file mmc1.docx]

**Sex-dependent impact of *Il6* deficiency in Parkinson’s Disease mice**

Fangzheng Chen^1,*^, Yufei Duan^1,*^, Mengze Wang^1^, Zhaolin Liu^1^, Jiayin Zhao^1^, Guangchun Fan^1^, Yongtao He^1^, Hongtian Dong^1^, Xiaoshuang Zhang^1^, Rong Fang^1^, Yunhe Zhang^1^, Xin Yan^1^, Chenye Shen^1^, Xiwen Tang^1^, Yuanyuan Ma^1^, Mei Yu^1^, Renyuan Zhou^1, #^, Jian Fei^2, 3, #^, Fang Huang^1, #^

* These authors contribute equally.

^1^ Department of Translational Neuroscience, Department of Urology, Jing’an District Centre Hospital of Shanghai; State Key Laboratory of Brain Function and Disorders and MOE Frontiers Center for Brain Science, Institutes of Brain Science, Fudan University, 138 Yixueyuan Road, Shanghai 200032, China.

^2^ School of Life Science and Technology, Tongji University, 1239 Siping Road, Shanghai 200092, China.

^3^ Shanghai Engineering Research Center for Model Organisms, Shanghai Model Organisms Center, INC., Pudong, Shanghai 201203, China.

^#^ Correspondence to: Dr. Renyuan Zhou, Department of Urology, Jing’an District Central Hospital, Fudan University, 259 Xikang Road, Shanghai 200040, China. E-mail: zhourenyuan@189.cn; Dr. Jian Fei, School of Life Sciences and Technology, Tongji University, 1239 Shipping Road, Shanghai 200092, China. E-mail: [jfei@tongji.edu.cn](mailto:jfei@tongji.edu.cn); or Dr. Fang Huang, State Key Laboratory of Medical Neurobiology, Shanghai Medical College, Fudan University, 138 Yixueyuan Road, Shanghai 200032, China. Phone: 86-21-54237856, Fax: 86-21-64174579, E-mail: huangf@shmu.edu.cn

E-mail information: Fangzheng Chen: 23211520003@m.fudan.edu.cn ; Yufei Duan: rebecca632321334@163.com ; Mengze Wang: 21301050151@m.fudan.edu.cn ; Zhaolin Liu: 17111520004@fudan.edu.cn ; Jiayin Zhao: 20211520024@fudan.edu.cn , Guangchun Fan:23111520010@m.fudan.edu.cn ; Yongtao He: 19111520002@fudan.edu.cn ; Hongtian Dong:18211520006@fudan.edu.cn ; Xiaoshuang Zhang: 18111520012@fudan.edu.cn ; Rong Fang:19211520013@fudan.edu.cn ; Yunhe Zhang: 22211520055@fudan.edu.cn ; Xin Yan: 23211520042@m.fudan.edu.cn ; Chenye Shen: 19111520026@fudan.edu.cn ; Xiwen Tang: 23211520033@m.fudan.edu.cn ; Yuanyuan Ma: mayuanyuan@fudan.edu.cn ; Mei Yu: yumei@fudan.edu.cn

**Running title:** Sex-dependent impact of *Il6* deficiency in PD

# Supplementary Information


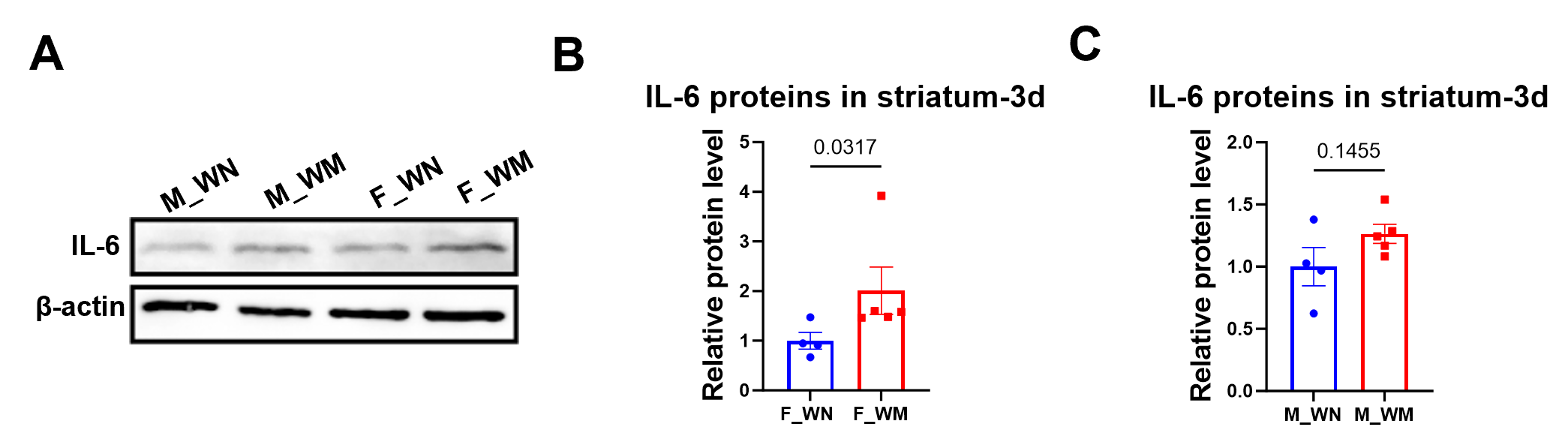


**Fig. S1 Striatal IL-6 protein expression in male and female WT mice at 3 days** **after MPTP administration.** (A-C) IL-6 protein levels in the striatum of both female and male mice at 3 days after MPTP administration; β-actin served as the loading control. n=4-5. F_WN (female WT saline group), F_WM (female WT MPTP group); M_WN (male WT saline group), M_WM (male WT MPTP group). Data were analyzed by T test for comparisons between groups. **p*<0.05

| **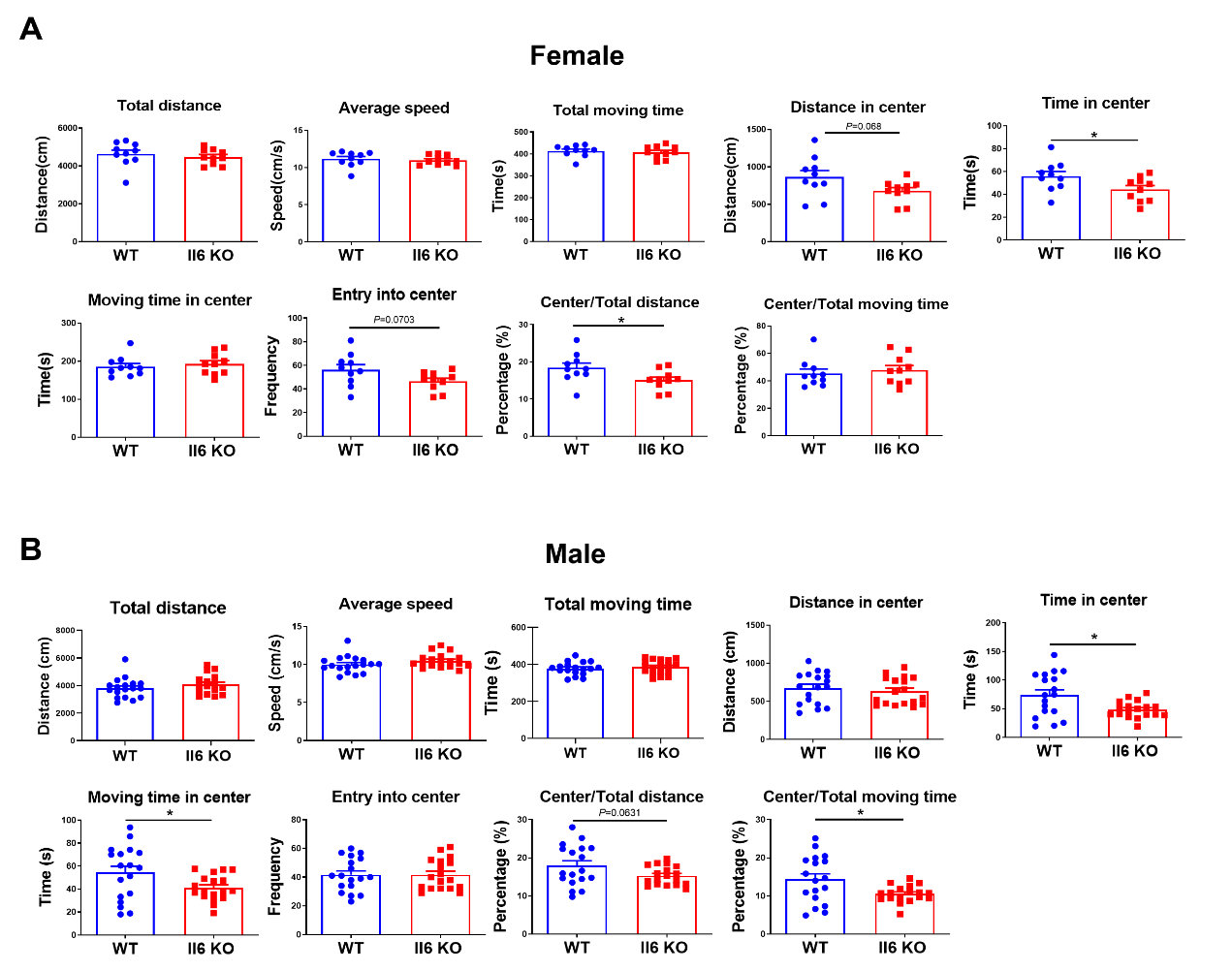** |
| --- |

**Fig. S2 The results of the Open field test in WT and *Il6* KO mice.** (A) Results of female mice. n=10. (B) Results of male mice. n=18. Data were analyzed by T test for comparisons between groups. **p*<0.05.

**
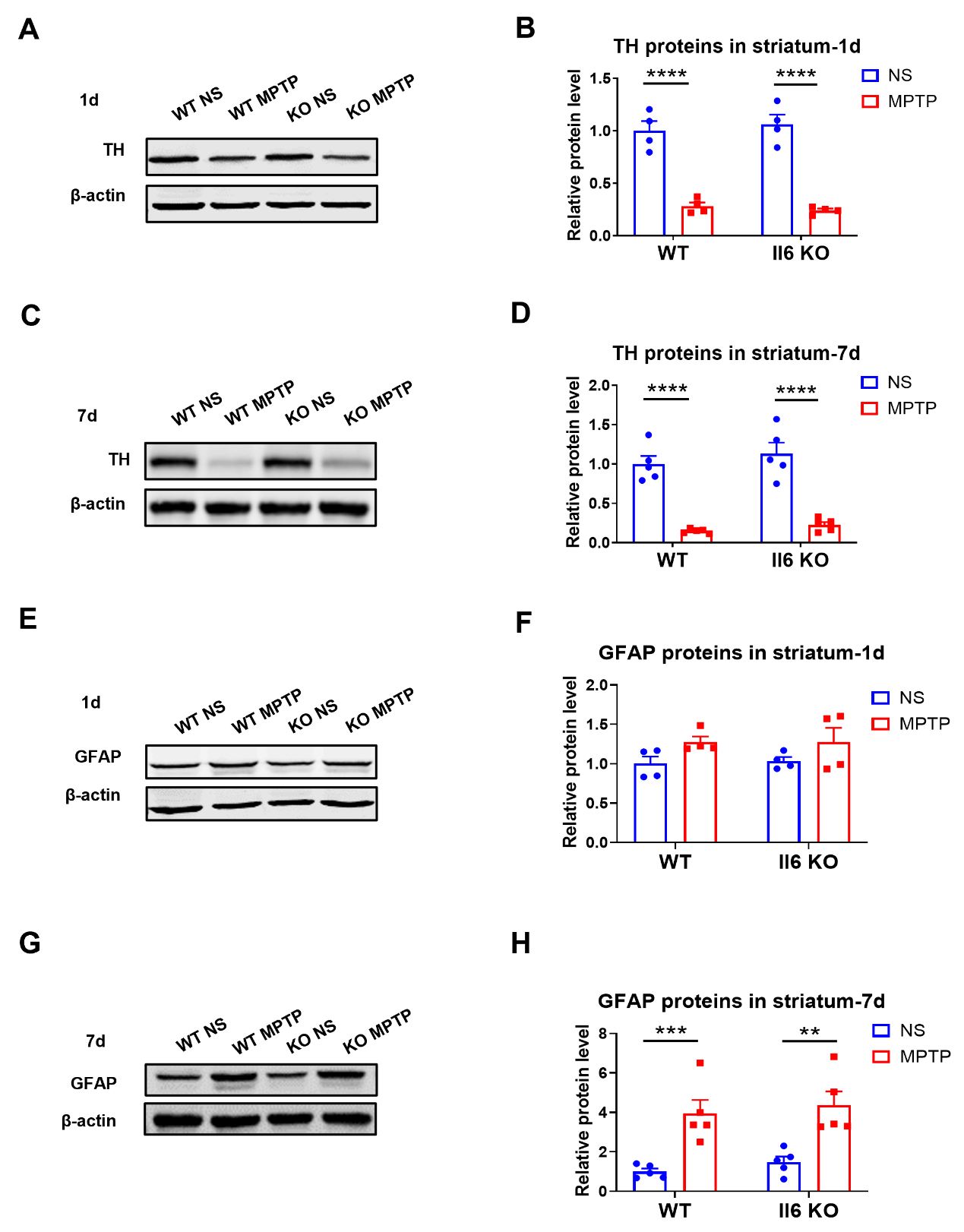
**

**Fig. S3 Striatal TH and GFAP protein expression in male *Il6* KO and WT mice after PD modeling.** (A, B) TH protein levels in the striatum of mice at 1 day after MPTP administration; β-actin served as the loading control. n=4. (C, D) TH protein levels in the striatum of mice at 7 days after MPTP administration; β-actin served as the loading control. n=5. (E, F) GFAP protein levels in the striatum of mice at 1 day after MPTP administration; β-actin served as the loading control; n=4. (G, H) GFAP protein levels in the striatum of mice at 7 days after MPTP administration; β-actin served as the loading control; n=5. Data were analyzed by Two-way ANOVA followed by Fisher’s LSD test for comparisons between groups. ***p*<0.01, ****p*<0.001. *****p*<0.0001.

| **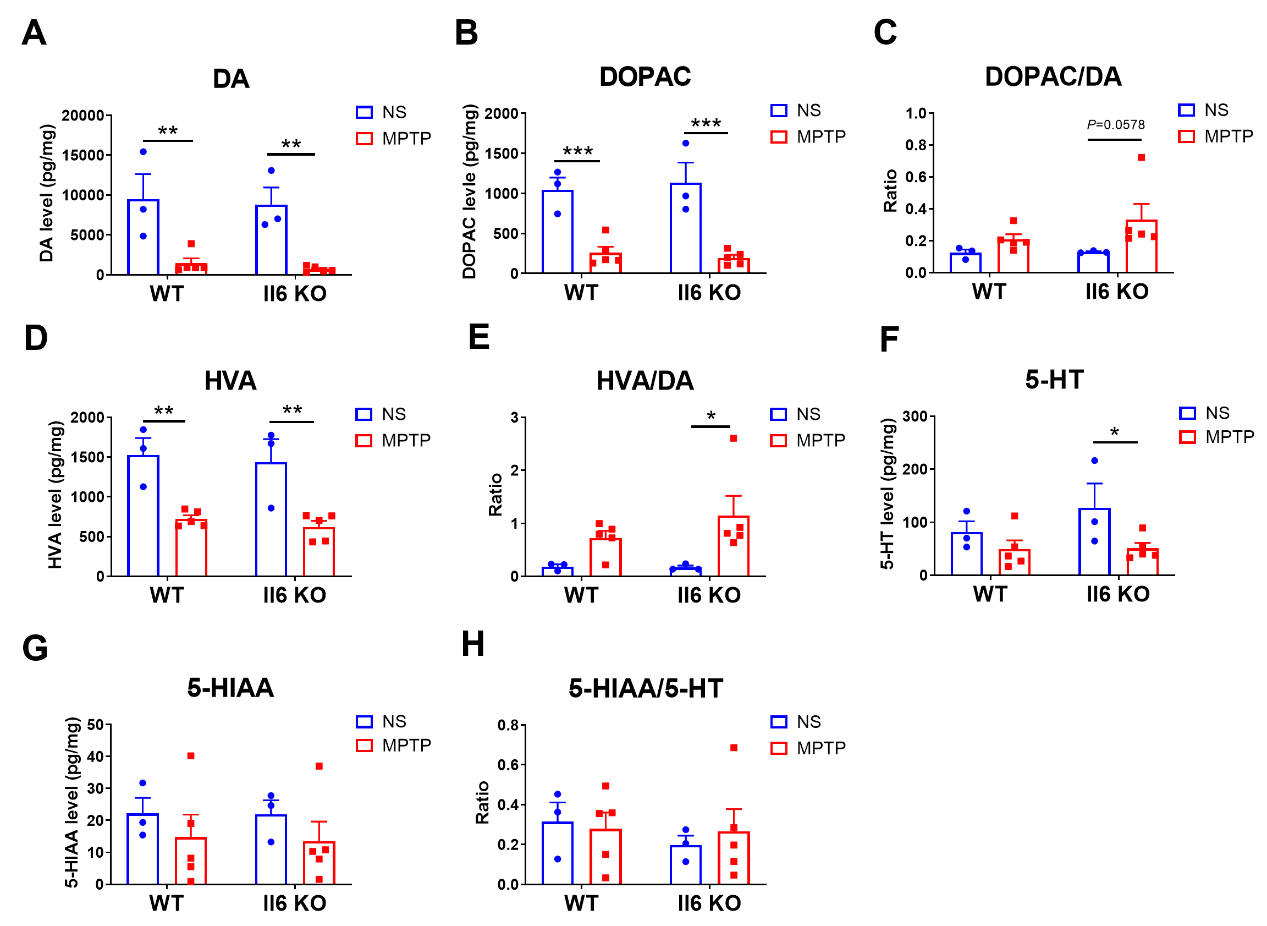** |
| --- |

**Fig. S4 Striatal levels of monoamine neurotransmitters and their metabolites, and turnover rates in male *Il6* KO and WT mice at 3 days after MPTP administration.** (A) DA; (B) DOPAC; (C) DOPAC/DA; (D) HVA; (E) HVA/DA; (F) 5-HT; (G) 5-HIAA; (H) 5-HIAA/5-HT. n=3-5. Data were analyzed by Two-way ANOVA followed by Fisher’s LSD test for comparisons between groups. **p*<0.05, ***p*<0.01, ****p*<0.001.

| **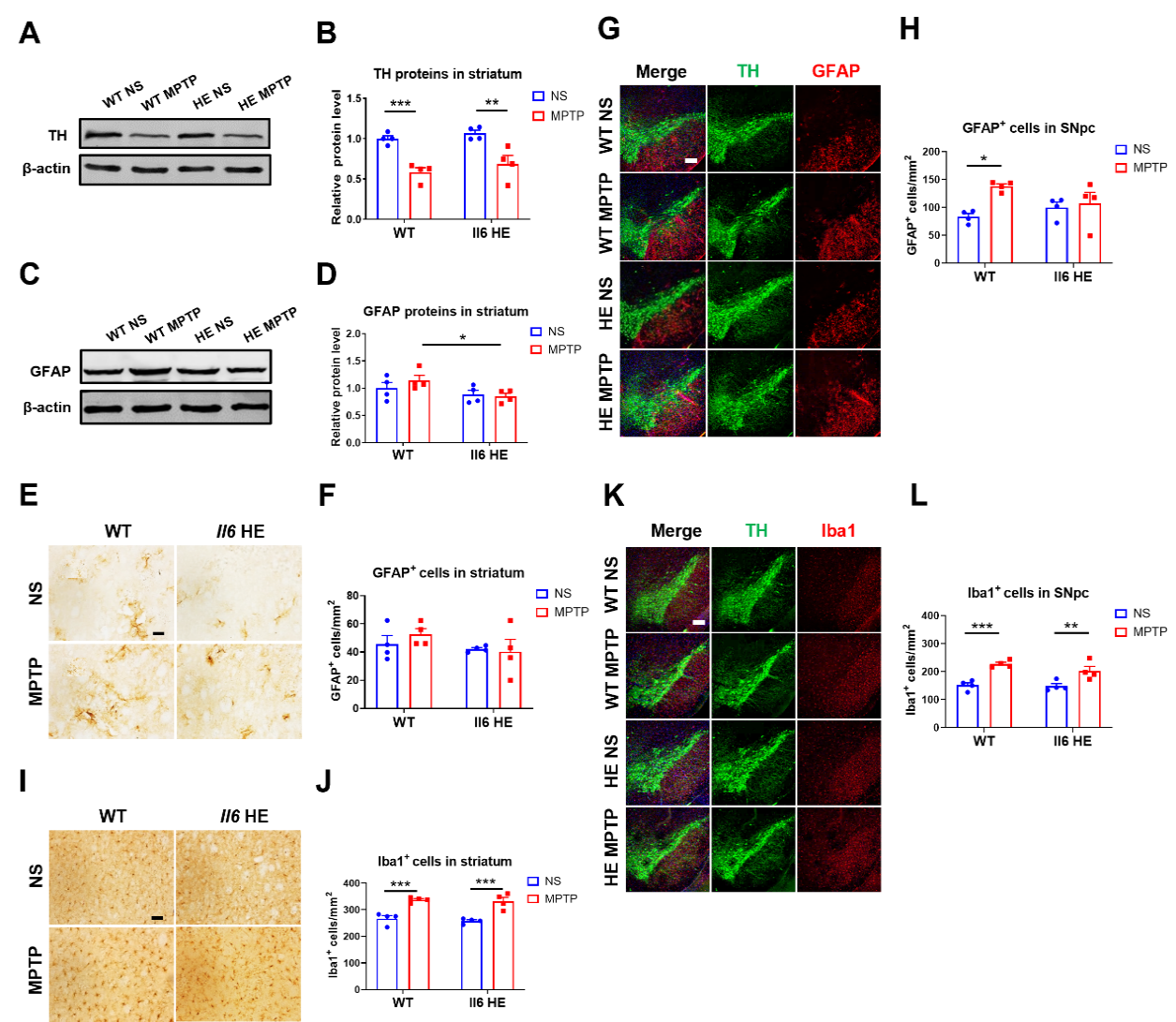** |
| --- |

**Fig. S5 Analysis of the striatal TH protein expression and the glial activation in the nigrostriatal pathway of male WT and *Il6* heterozygous mice at 1 day after MPTP administration.** (A, B) TH protein levels in the striatum. n=4. (C, D) GFAP protein levels in the striatum. n=4. (E, F) Immunohistochemical staining and quantification of GFAP-positive cells in the striatum. n=4. Scale bar 50 μm. (G, H) Immunofluorescence staining and quantification of GFAP-positive cells in the SNpc. Representative TH (green) and GFAP (red) staining images are shown. Scale bar 200 μm. (I, J) Immunohistochemical staining and quantification of Iba1-positive cells in the striatum. n=4. Scale bar 50 μm. (K, L) Immunofluorescence staining and quantificaiton of Iba1-positive cells in the SNpc. Representative TH (green) and GFAP (red) staining images are shown. n=4. Scale bar 200 μm. Data were analyzed by Two-way ANOVA followed by Fisher’s LSD test for comparisons between groups. **p*<0.05, ***p*<0.01, and ****p*<0.001.

| **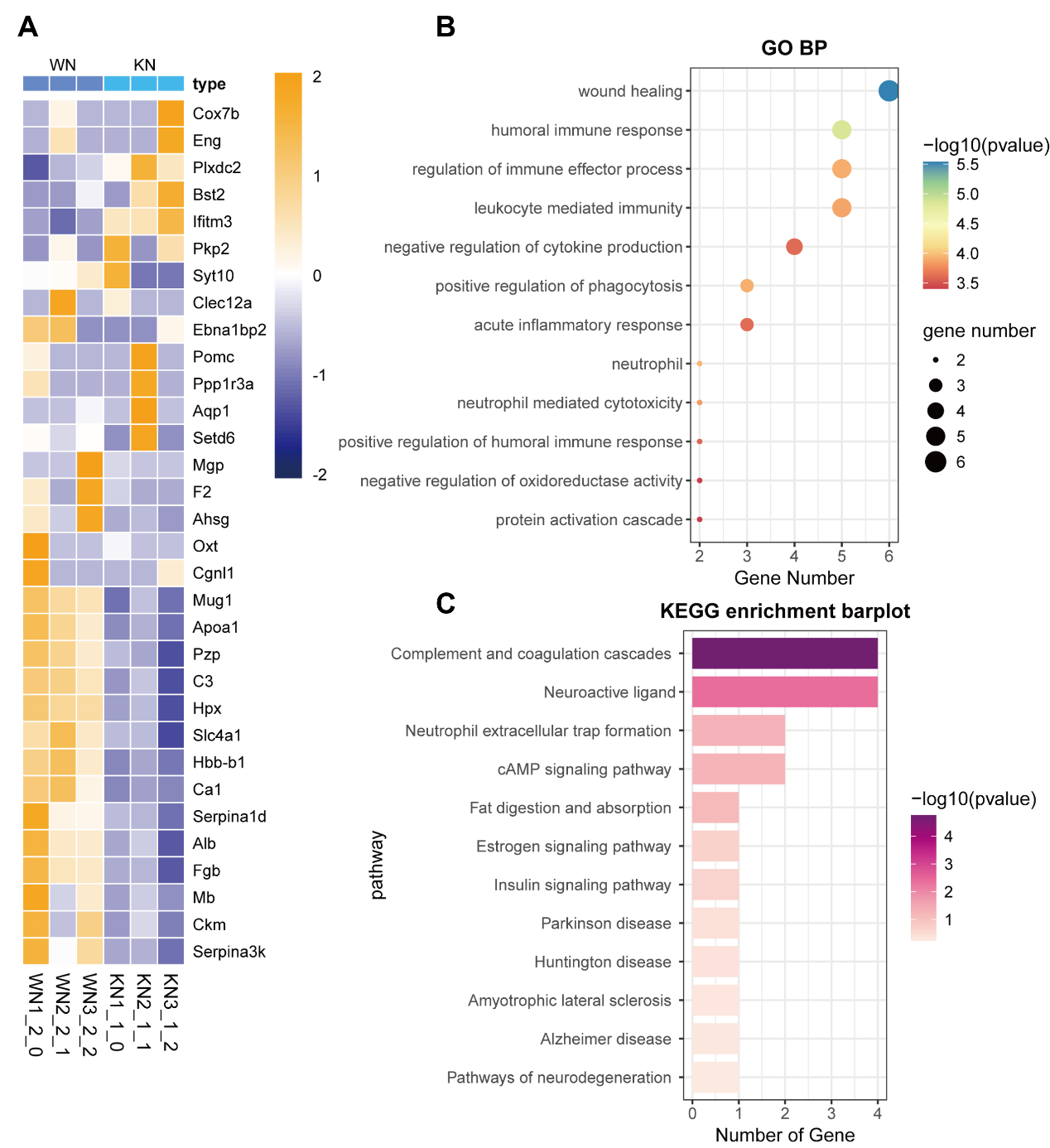** |
| --- |

**Fig. S6 Analysis of differentially expressed proteins in the striatum of female *Il6* KO mice and WT mice under physiological conditions.** (A) Heatmap analysis. (C) GO analysis. (D) KEGG analysis. WN: WT NS; KN: KO NS.

| 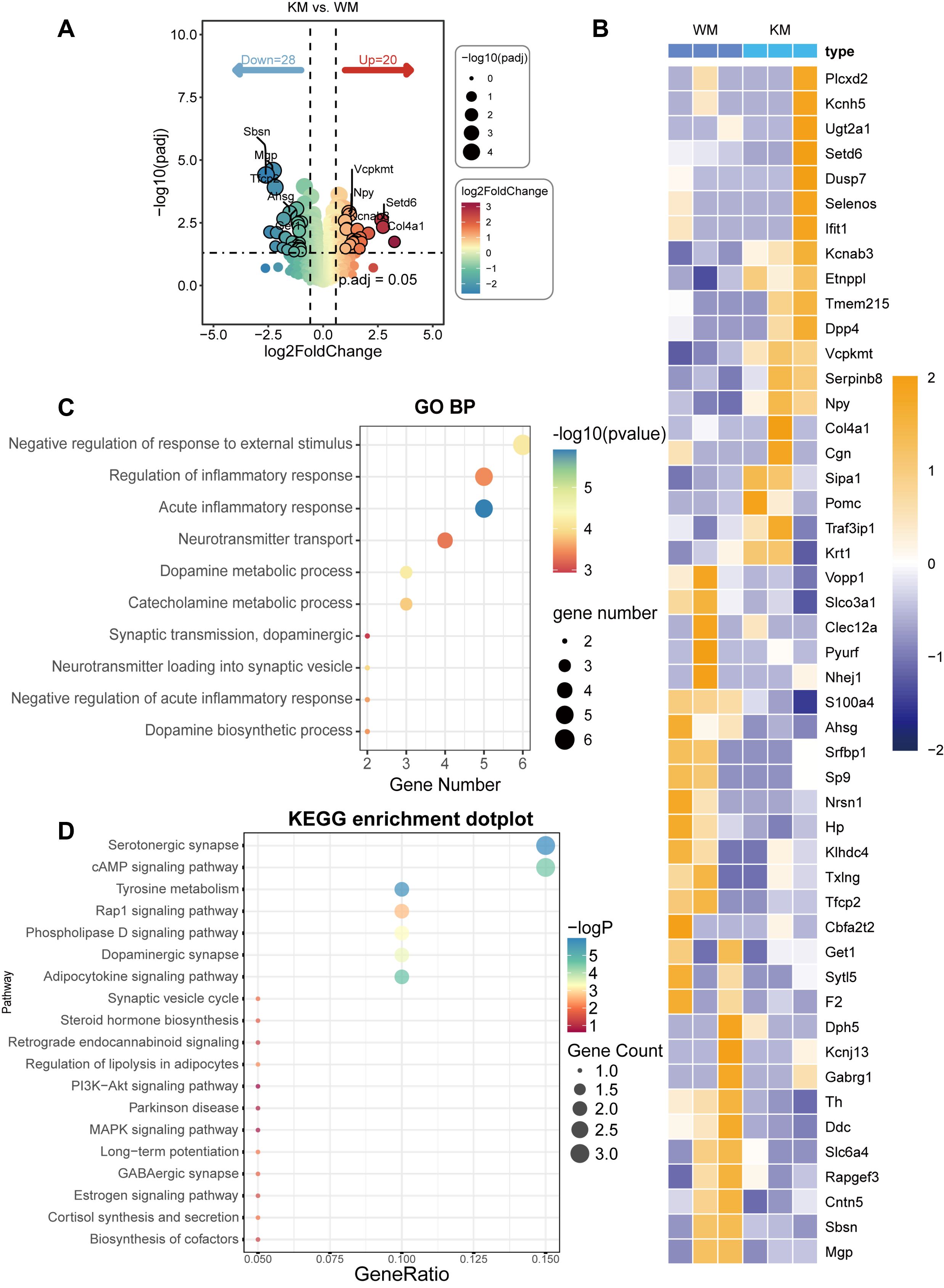 |
| --- |

**Fig. S7 Analysis of differentially expressed proteins in the striatum of female *Il6* KO mice and WT mice at 3 days after MPTP administration.** (A) Volcano Plot. (B) Heatmap analysis. (C) GO analysis. (D) KEGG analysis. WM: WT MPTP; KM: KO MPTP.

| **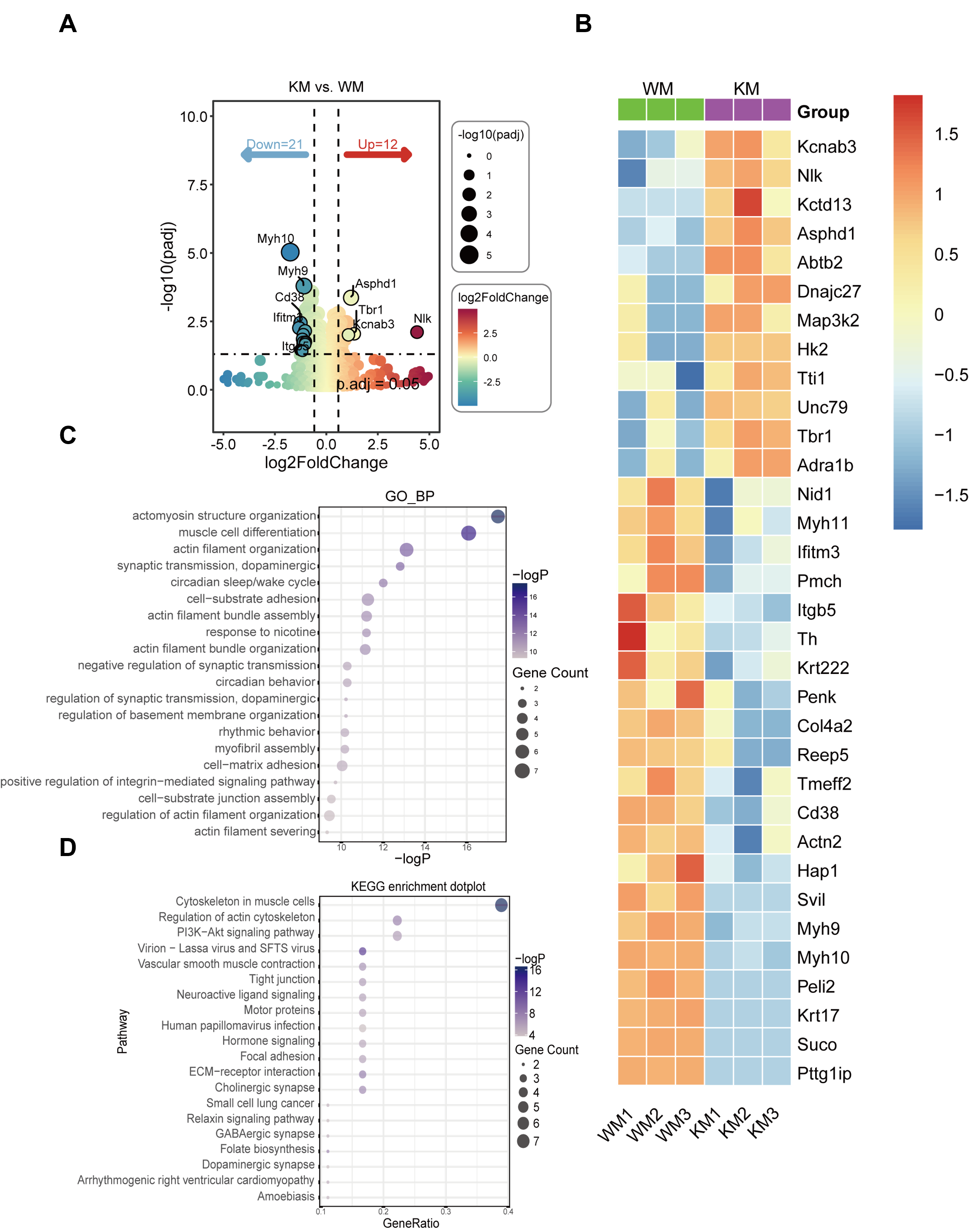** |
| --- |

**Fig. S8 Analysis of differentially expressed proteins in the striatum of male *Il6* KO mice at 3 days after MPTP administration.** (A) Volcano Plot. (B) Heatmap analysis. (C) GO analysis. (D) KEGG analysis. WM: WT MPTP; KM: KO MPTP.

| 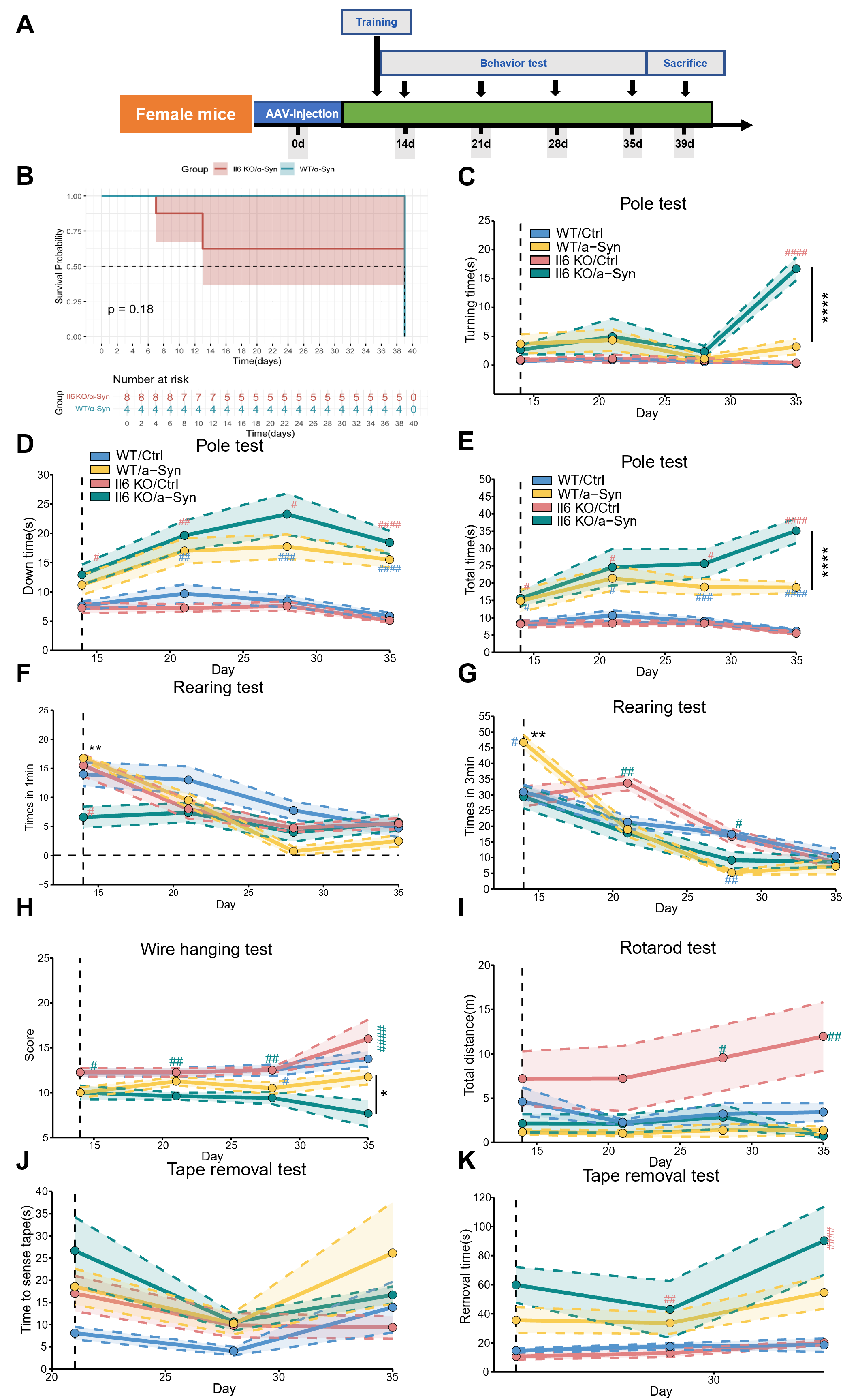 |
| --- |

**Fig. S9** **The experimental schedule and the results of behavioral tests in female WT and *Il6* KO** **mice following AAV α-Synuclein^A53T^ injection.** (A) The flowchart of chronic AAV α-Synuclein^A53T^-induced PD mice model and PD-related behavioral tests conducted in female *Il6* KO mice and WT mice. (B) The survival curve of chronic AAV α-Synuclein^A53T^-induced female PD mice. (C-E) The results of the Pole test. Turning time (C), down time (D), and total time (E) were shown. (F-G) The total rearing times in 1 or 3 minutes of the Rearing test. (H) The scores in the Wire hanging test. (I) The total moving distance in the Rotarod test. (J-K) The time to sense the tape and removal time in the Tape removal test. Data were analyzed by Two-way RANOVA followed by Fisher’s LSD test for comparisons between groups. n=3-4. Control vs α-Synuclein: ^#^*p*<0.05, ^##^*p*<0.01 and ^####^*p*<0.0001; WT vs *Il6^-/-^*: **p*<0.05, ***p*<0.01 and *****p*<0.0001.

| 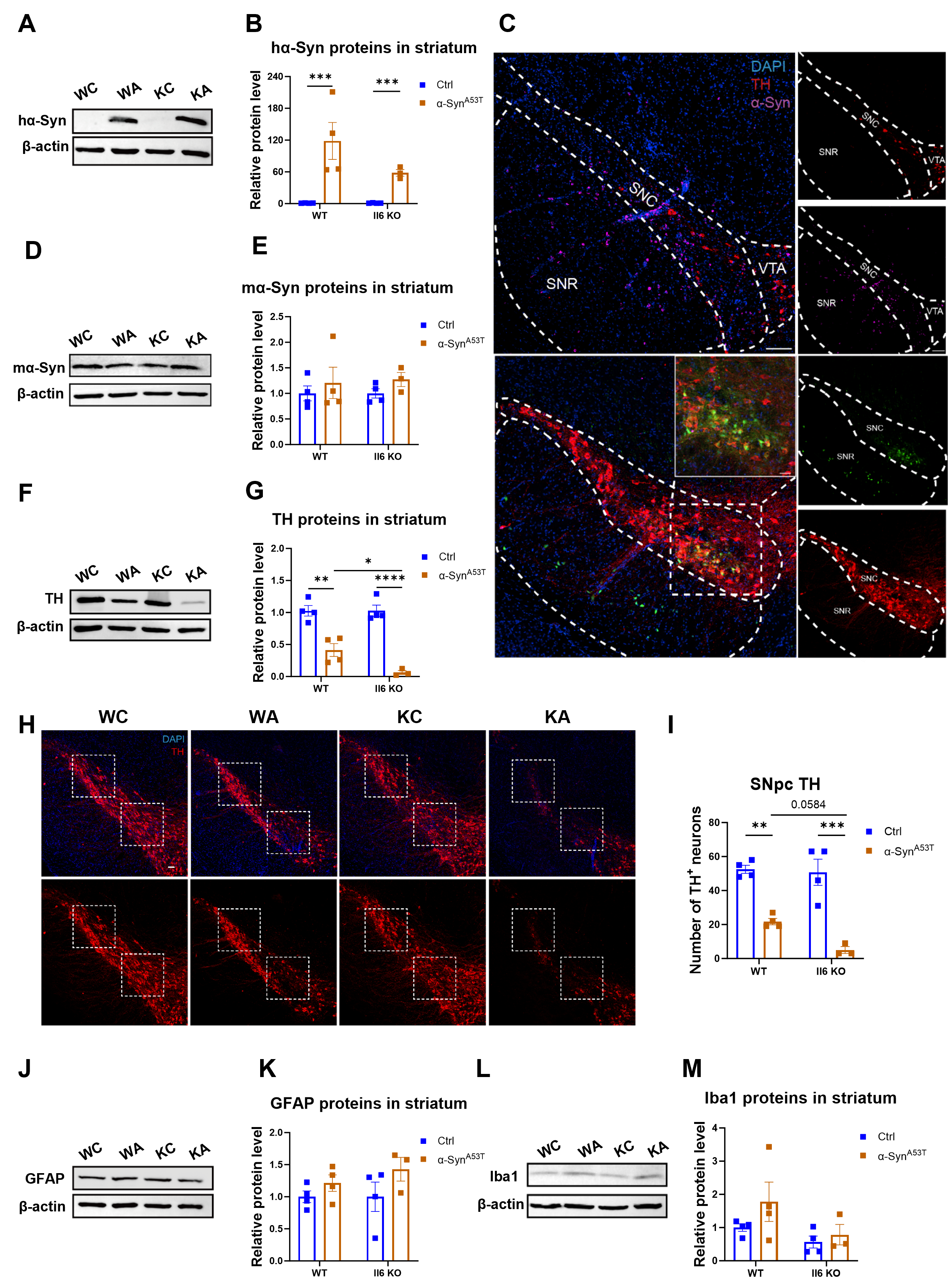 |
| --- |

**Fig. S10** **Analysis of the dopaminergic system and neuroinflammation in the nigrostriatal pathway of female WT and *Il6* KO mice following AAV α-Synuclein^A53T^ injection.** (A, B) The striatal levels of human α-Synuclein proteins; β-actin served as the loading control; (C) Immunofluorescence staining for tyrosine hydroxylase (TH, red), α-Synuclein (violet) and GFP in the SN region after injection of AAV α-Synuclein^A53T^ (up) or AAV GFP (bottom) respectively. (D, E) The striatal levels of mouse α-Synuclein proteins; β-actin served as the loading control. (F, G) The striatal levels of TH proteins; β-actin served as the loading control. (H, I) Immunofluorescence staining for TH (red) in the SN region (H) and quantification of TH-positive cells (I). (J, K) The striatal levels of GFAP proteins; β-actin served as the loading control. (L, M) The striatal levels of Iba1 proteins; β-actin served as the loading control. Data were analyzed by Two-way ANOVA followed by Fisher’s LSD test for comparisons between groups. n=3-4. Scale bar: 100 μm and 50 μm (zoom). ***p*<0.01, and ****p*<0.001.

**
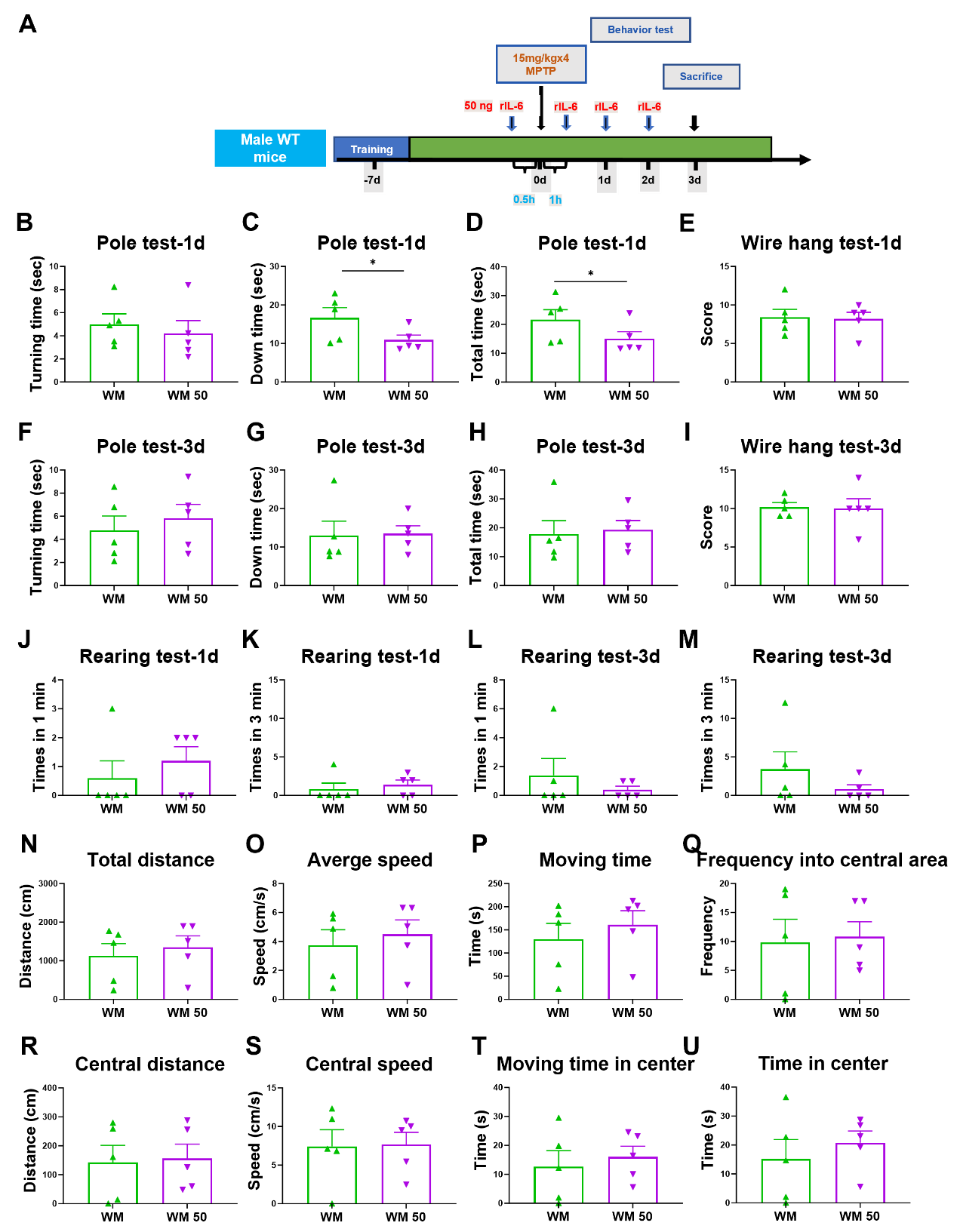
**

**Fig. S11** **The results of behavioral tests in MPTP-challenged WT and *Il6* KO mice with a dose of 50 ng rIL-6 intervention.** (A) Flowchart of PD modeling and rIL-6 treatment. (B-E) Results of the Pole test (B-D), Wire hanging test (E), and Rearing test (J, K) in WT mice one day after MPTP administration. (F-I) Results of the Pole test (F-H), Wire hanging test (I), and Rearing test (L, M) in WT mice at 3 days after MPTP administration. (N-U) Results of the Open field test in WT mice at 3 days after MPTP administration. Representative total moving distance (N), average speed (O) total moving time (P), frequency into central area (Q), central distance (R), central speed (S), moving time in center (T) and time in center (U) are shown; n=5. Mice in WM group were the same cohort as WM in Fig.6. Data were analyzed by T test for comparisons between groups. **p*<0.05.

**
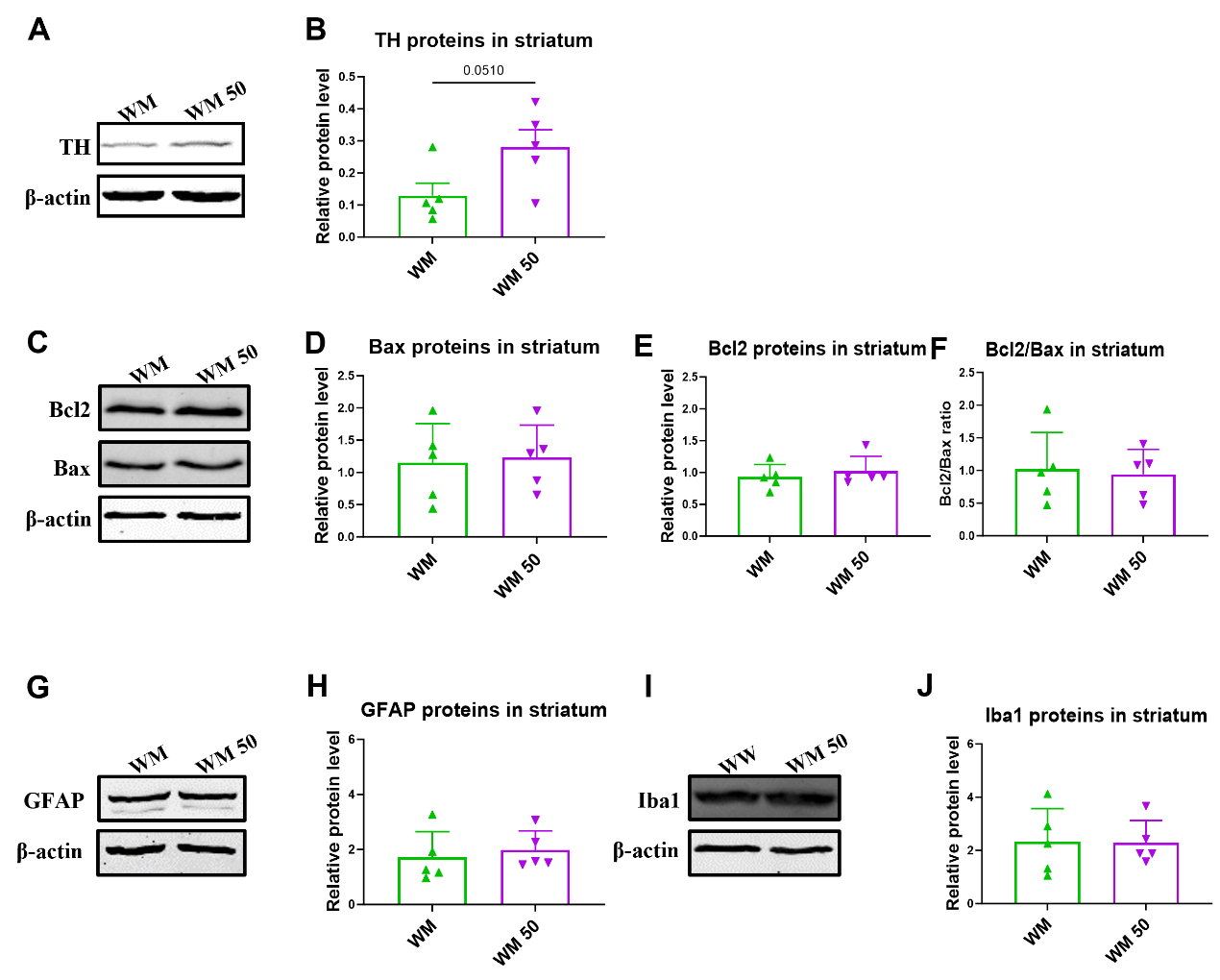
**

**Fig. S12** **The striatal TH protein expression in MPTP-challenged WT with a dose of 50 ng rIL-6 intervention.** (A, B) Representative Western blot images and quantification of striatal TH proteins in WT mice (n=5) at 3 days post-MPTP administration. β-actin served as the loading control. (C-F) Representative Western blot images and quantification of striatal Bcl2 and Bax proteins in WT mice (n=5) at 3 days post-MPTP administration. β-actin served as the loading control. (G-J) Representative Western blot images and quantification of striatal GFAP and Iba1 proteins in WT mice (n=5) at 3 days post-MPTP administration. β-actin served as the loading control. Mice in WM group were the same cohort as WM in Fig.6. Data were analyzed by T test for comparisons between groups.

**
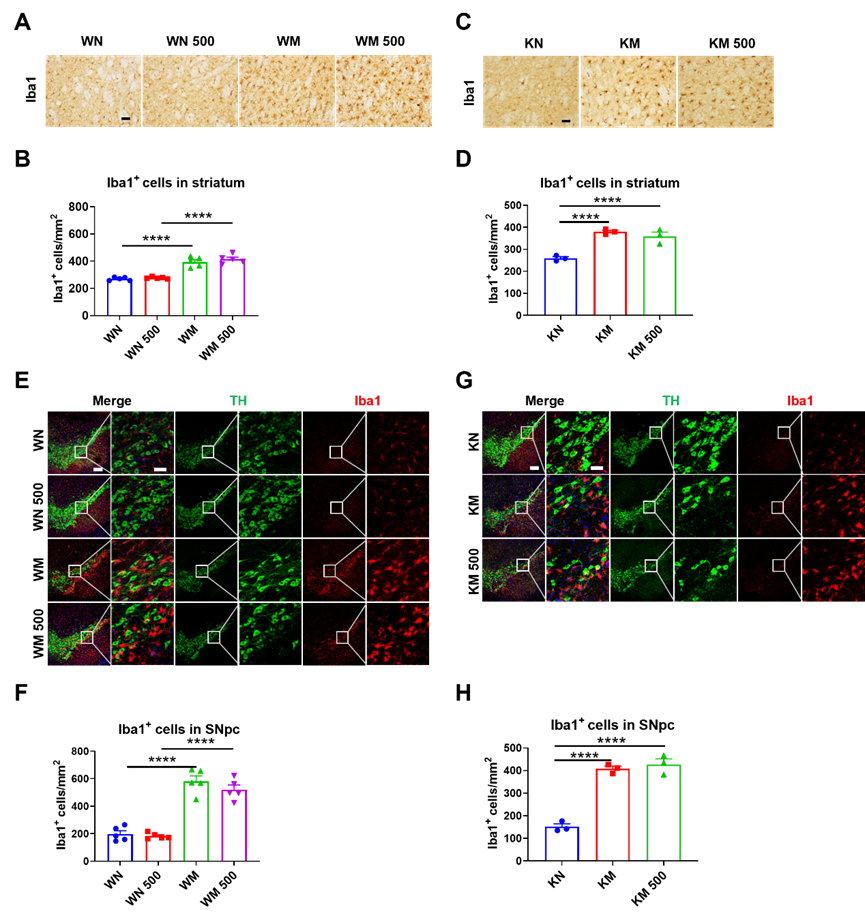
**

**Fig. S13 Microglial activation in the nigrostriatal pathway of MPTP-challenged WT and *Il6* KO mice with rIL-6 intervention.** (A-D) Immunohistochemical staining and cell counting of Iba1-positive cells in the striatum of WT mice (A, B) (n=5) and *Il6* KO mice (C, D) (n=3) at 3 days after MPTP administration. Scale bar 50 μm (zoom). (E-H) Immunofluorescence staining and cell counting of Iba1-positive cells in the SNpc of WT mice (E, F) (n=5) and *Il6* KO mice (G, H) (n =3) at 3 days after MPTP administration. Representative TH (green) and Iba1 (red) images are shown. Scale bar: 200 μm and 50 μm (zoom). *****p*<0.0001.

**
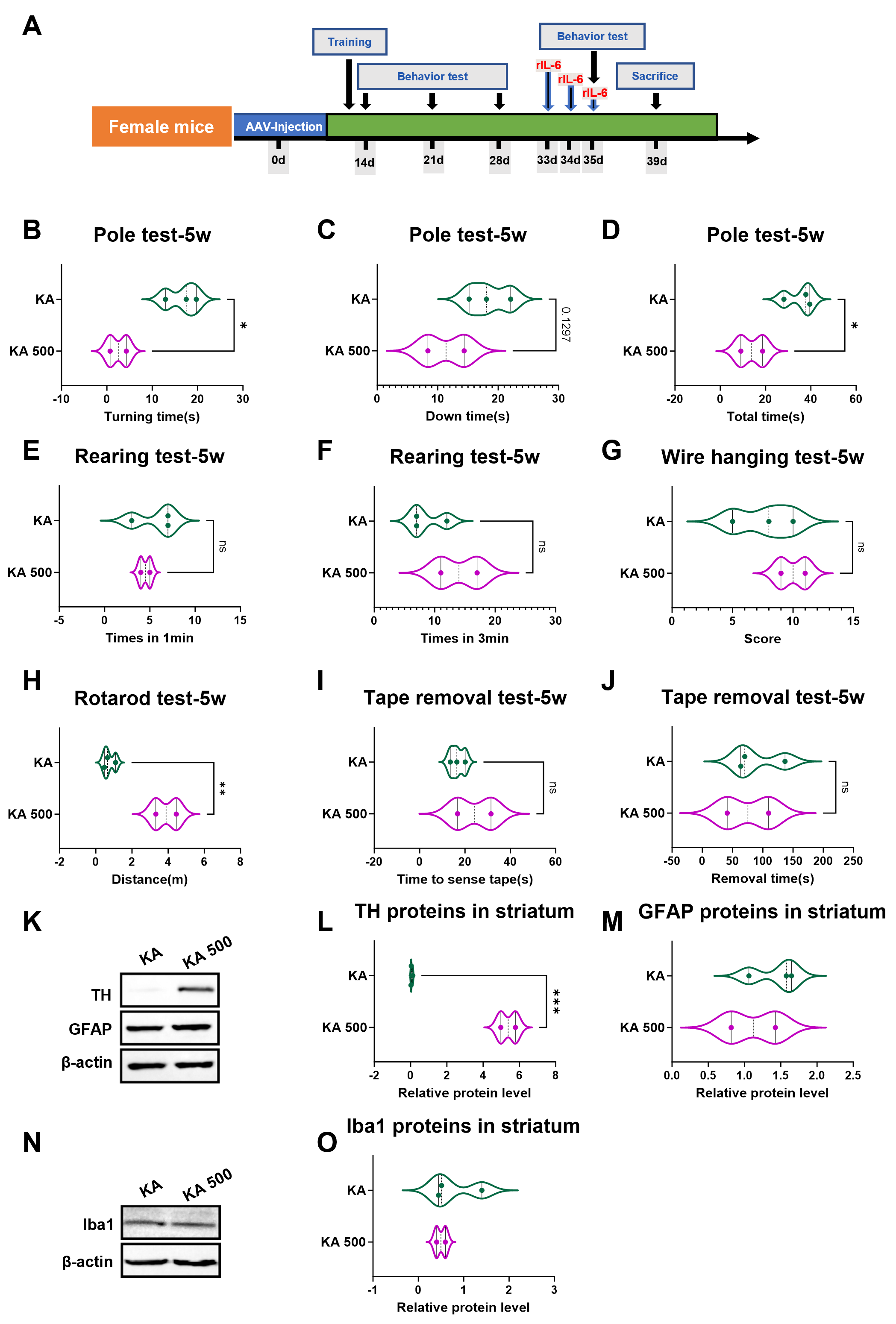
**

**Fig. S14** **The results of behavioral tests and the analysis of striatal TH, GFAP and Iba1 protein expression in α-Synuclein^A53T^-induced *Il6* KO PD mice with or without rIL-6 intervention.** (A) The flowchart of PD modeling and rIL-6 treatment. (B-J) The result of the Pole test (B-D), Rearing test (E, F), Wire hanging test (G), Rotarod test (H), and Tape removal test (I, J) in *Il6* KO mice 35 days after α-Synuclein injection. (K-O) Representative Western blot images and quantification of striatal TH, GFAP and Iba1 proteins in *Il6* KO mice (n = 2-3); β-actin served as the loading control. Mice in KA group were the same cohort as KA in Fig. S10. Data were analyzed by T test for comparisons between groups. **p*<0.05, ***p*<0.01, ****p*<0.001.

**
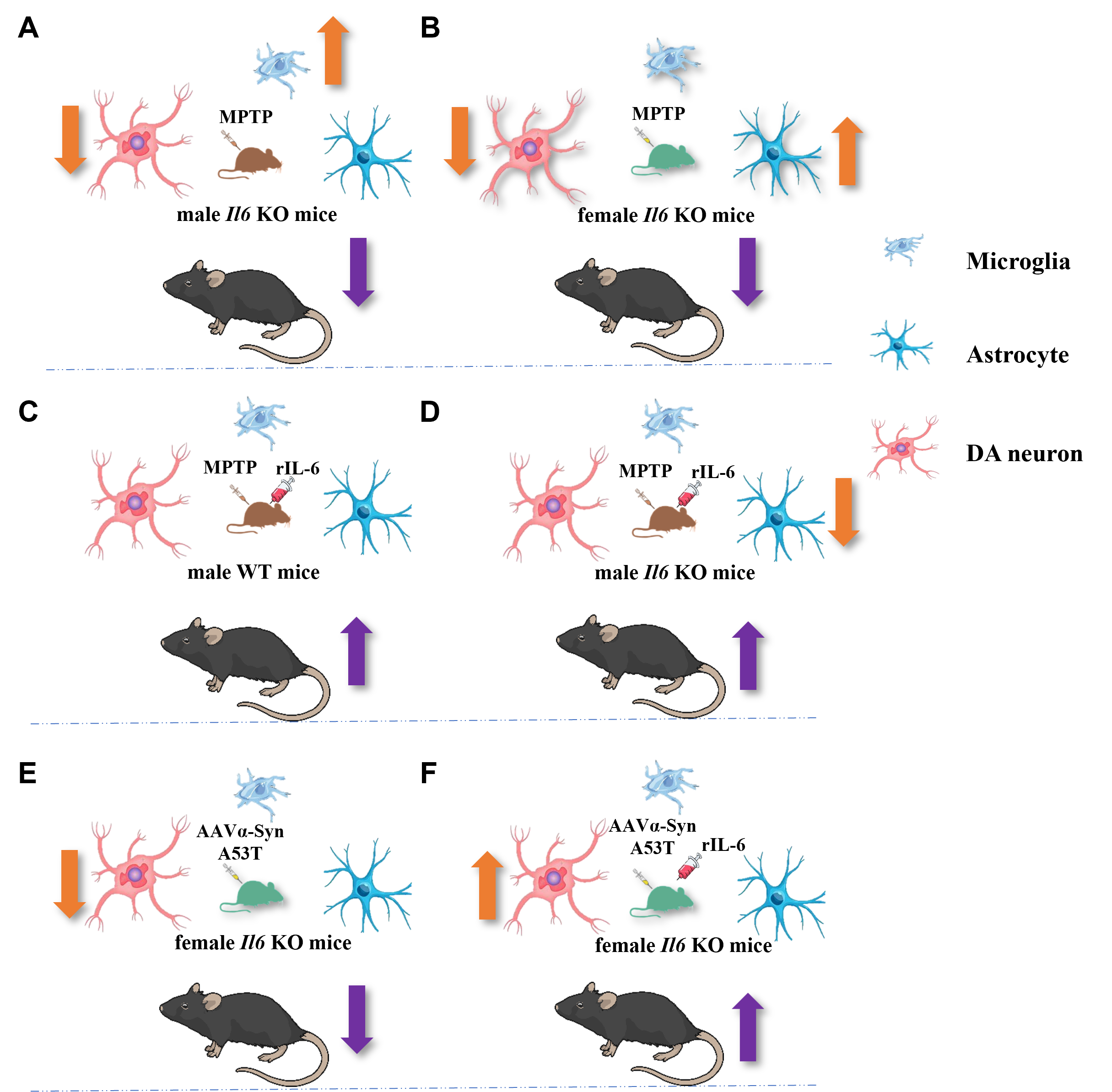
**

**Fig. S15 Summary of the effects of IL-6 on PD pathology.** (A) Effects of *Il6* deletion on MPTP-induced PD pathology in male mice. (B) Effects of *Il6* deletion on MPTP-PD pathology in female mice. (C) Effects of rIL-6 intraperitoneal administration in male WT PD mice. (D) Effects of rIL-6 intraperitoneal administration in male KO PD mice. (E) Effects of *Il6* deletion on α-Synuclein^A53T^-induced (α-Syn^A53T^) PD pathology in female mice. (F) Effects of rIL-6 intraperitoneal administration in female KO PD mice. ↑, stimulation; ↓, inhibition.
